# Update on left ventricular outflow tract obstruction

**DOI:** 10.1186/s44348-025-00049-2

**Published:** 2025-07-01

**Authors:** Jae-Kwan Song, Byung Joo Sun, Dae-Hee Kim, Sung Ho Jung

**Affiliations:** 1 Department of Cardiology, Asan Medical Center, University of Ulsan College of Medicine, Seoul, Republic of Korea; 2 Department of Cardiothoracic Surgery, Asan Medical Center, University of Ulsan College of Medicine, Seoul, Republic of Korea

**Keywords:** Hypertrophic cardiomyopathy, Left ventricular outflow tract obstruction, Echocardiography

## Abstract

The diagnosis and management of left ventricular outflow tract obstruction have evolved substantially since its initial characterization by invasive cardiac catheterization nearly 70 years ago. This review traces the historical progression of our understanding of left ventricular outflow tract obstruction, with a particular focus on the pivotal role of echocardiography in redefining its detection, hemodynamic assessment, and clinical relevance, especially as its prevalence is expected to increase along with the growing aging population.

**Supplementary Information:**

The online version contains supplementary material available at 10.1186/s44348-025-00049-2.

## Background

**Fig. 1:**
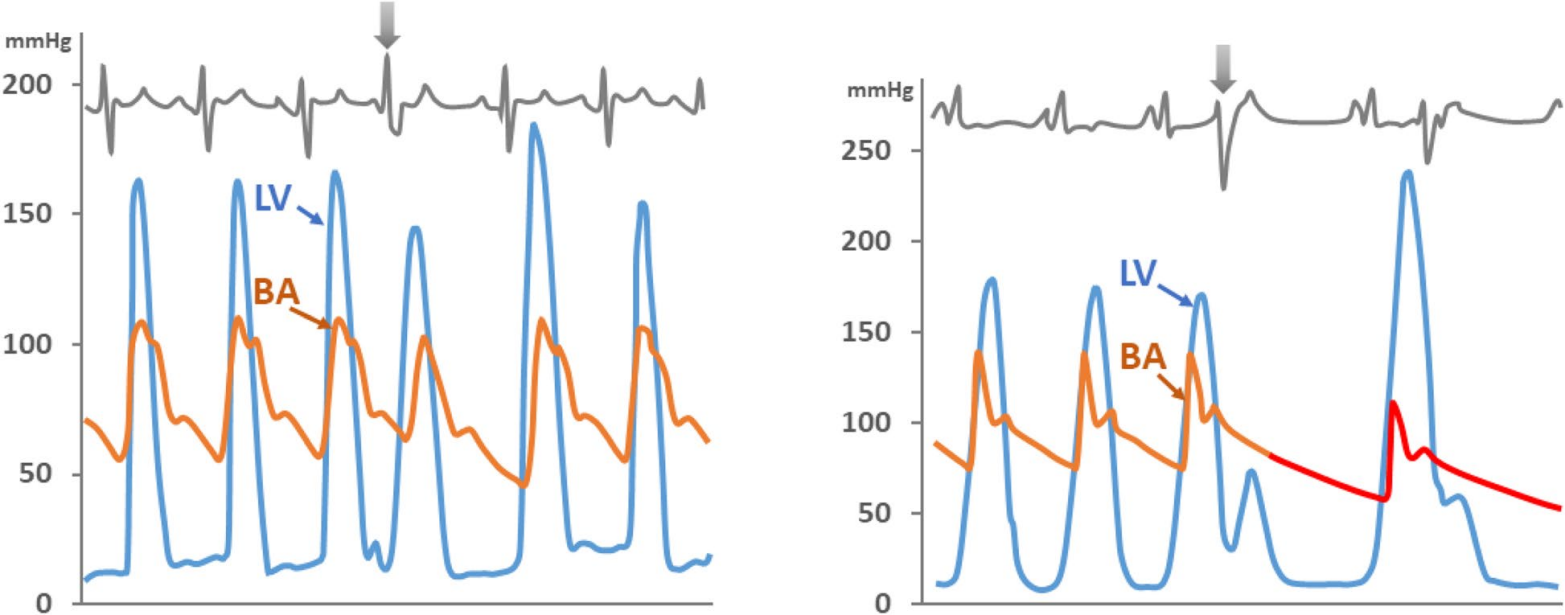
Representative pressure tracings for differential diagnosis between discrete valvular stenosis and idiopathic hypertrophic subaortic stenosis. **A** In the presence of valvular aortic stenosis or discrete subvalvular stenosis characterized by fixed stenosis, arterial pressure does not change after a premature beat, whereas **B** a decrease in arterial pressure is prominent after a premature beat in idiopathic hypertrophic subaortic stenosis. This inverse change in arterial pressure compared with left ventricular pressure after a premature beat has been an established differential point for more than 60 years. Modified from Brockenbrough et al. [4], with permission from Wolters Kluwer Health Inc

The first reports of a significant pressure gradient between the left ventricular main cavity and the left ventricular outflow tract (LVOT) identified during cardiac catheterization were published nearly 70 years ago [1, 2]. As this phenomenon was not associated with aortic valvular stenosis, various terms were used to describe this condition, including “functional obstruction of the left ventricular (LV),” “acquired aortic subvalvular stenosis,” and “idiopathic hypertrophic subaortic stenosis” [3, 4]. Subsequent clinical observations revealed that this pathological condition was characterized by massive LV hypertrophy not associated with any volume or pressure overload. Thus, the term “hypertrophic cardiomyopathy (HCM)” was used to distinguish it from secondary LV hypertrophy due to pressure or volume overload [5]. Initially, LVOT obstruction (LVOTO) was considered a pathognomonic finding of HCM, and cardiac catheterization for documenting the pressure gradient within the LV was deemed essential for an accurate diagnosis of HCM. The primary focus was on differentiating between fixed valvular aortic stenosis or discrete subvalvular stenosis and the dynamic nature of LVOTO in obstructive HCM using invasive cardiac catheterization (Fig. 1) [4]. However, LVOTO is often absent in patients with HCM. The noninvasive echo-Doppler technique has become crucial for accurate diagnosis of HCM and has led to better understanding of the mechanism of LVOTO in such patients [6, 7].

## Mechanisms of LVOTO

### Asymmetric septal hypertrophy and systolic anterior motion of the mitral valve

**Fig. 2:**
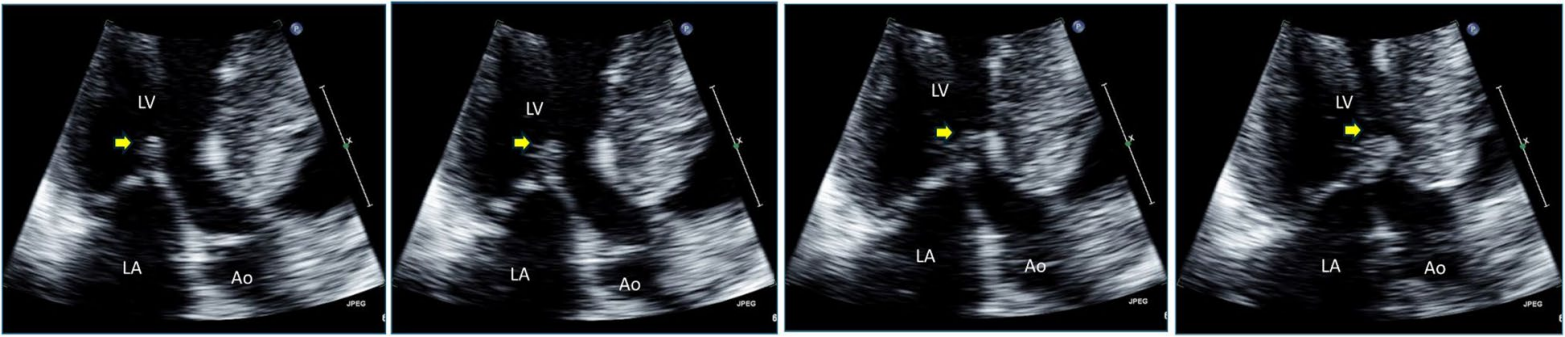
Two-dimensional echocardiographic images showing the mechanism of left ventricular outflow tract obstruction in hypertrophic cardiomyopathy (Video1). Frame-by-frame images clearly demonstrate systolic anterior motion of the mitral leaflet (arrows) resulting in septal contact and obstruction in the left ventricular outflow tract. Ao, aorta; LA, left atrium; LV, left ventricle

**Fig. 3:**
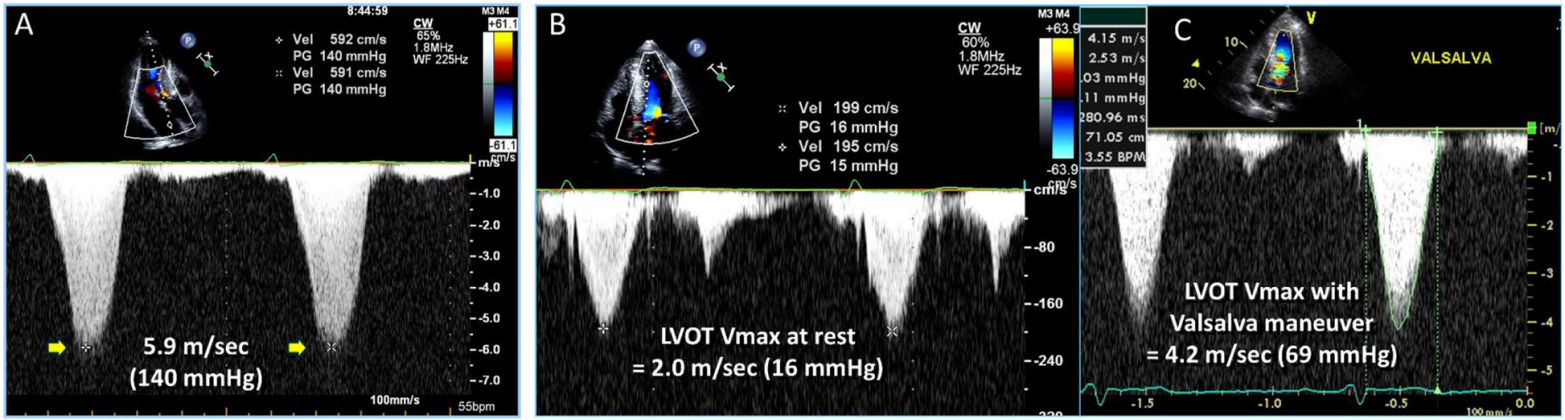
Representative Doppler tracings of resting and latent left ventricular outflow tract obstruction. **A** Markedly increased systolic peak velocity with a typical delayed peak and dagger shape is recorded at rest, suggestive of resting outflow tract obstruction. In some patients, (**B**) peak velocity at rest is not high but (**C**) increases significantly with the Valsalva maneuver, suggesting latent outflow tract obstruction

Early clinical experiences using noninvasive echocardiography demonstrated the characteristic asymmetric septal hypertrophy (ASH) and the mechanism of LVOTO. Echocardiography clearly illustrates that the systolic anterior motion of the mitral leaflet (SAM) contacting the thick interventricular septum is a primary mechanism of LVOTO (Fig. 2, Video 1) [6]. Continuous wave Doppler recordings of the LVOTO jet provide a distinctive Doppler signal with a delayed peak, resulting in a typical “dagger-shaped” systolic velocity profile (Fig. 3). The dynamic nature of LVOTO, due to SAM and septal contact, is well documented by the varying degrees of peak LVOTO velocity, which depends on the hemodynamic status. Thus, latent LVOTO, characterized by absent or negligible LVOTO at rest and severe LVOTO induced by the Valsalva maneuver or exercise, is another defining feature of obstructive HCM (Fig. 3) [8]. These echo-Doppler findings are widely used as diagnostic criteria for HCM and for quantifying LVOTO, which is defined as an instantaneous peak Doppler LVOT pressure gradient ≥ 30 mmHg at rest or during physiological provocation. A gradient ≥ 50 mmHg is the threshold at which LVOTO becomes hemodynamically significant [9, 10]. The initial surgical approach for ASH, including myectomy or myotomy, has resulted in significant hemodynamic improvement in patients with LVOTO. Consequently, septal reduction therapies targeting ASH, such as surgical myectomy and alcohol septal ablation, have been developed and established as standard therapeutic options for managing SAM-septal contact [9, 10].

HCM is characterized by marked heterogeneity in morphological types (obstructive vs. nonobstructive), genetic variations, and clinical manifestations. While the hallmark of HCM is increased LV wall thickness, alterations in the mitral valve and the subvalvular apparatus have been a topic of interest for more than four decades [11–13]. The initial hypothesis regarding the potential contribution of the mitral valve and the subvalvular apparatus to the development of significant LVOTO was proposed by pioneering hemodynamic and angiographic studies [14, 15]. The successful clinical introduction of noninvasive cardiac imaging modalities including echocardiography with Doppler techniques and cardiac magnetic resonance imaging (CMR) has been critical for increasing understanding of these abnormalities. These advancements have also contributed to the development of effective surgical approaches [16].

### Abnormalities in the size and position of the mitral valve

**Fig. 4:**
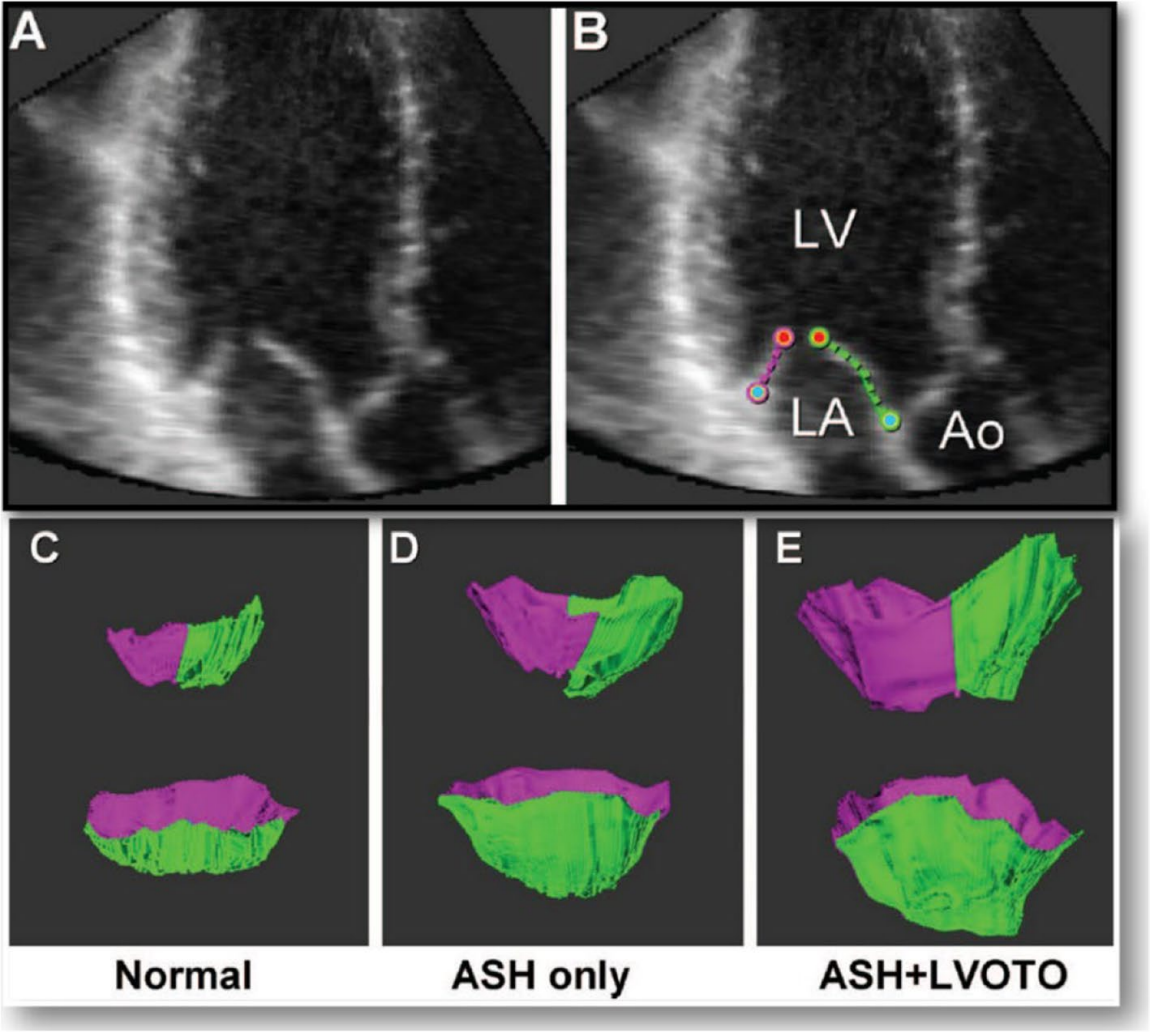
**A**, **B** Representative component view with diastolic leaflet traces for three-dimensional reconstruction of the mitral leaflet area calculation. **C**–**E** Representative open mitral leaflet area measurements (green and purple) for anterior and posterior leaflets viewed from the side, top row, lateral commissure in foreground, and left ventricular outflow tract (LVOT) aspect below, greatest asymmetrical septal hypertrophy (ASH) and ASH + LVOT obstruction. LA, left atrium; LV, left ventricle; Ao, aorta. Reproduced from Kim et al. [18], with permission from Wolters Kluwer Health Inc

**Fig. 5:**
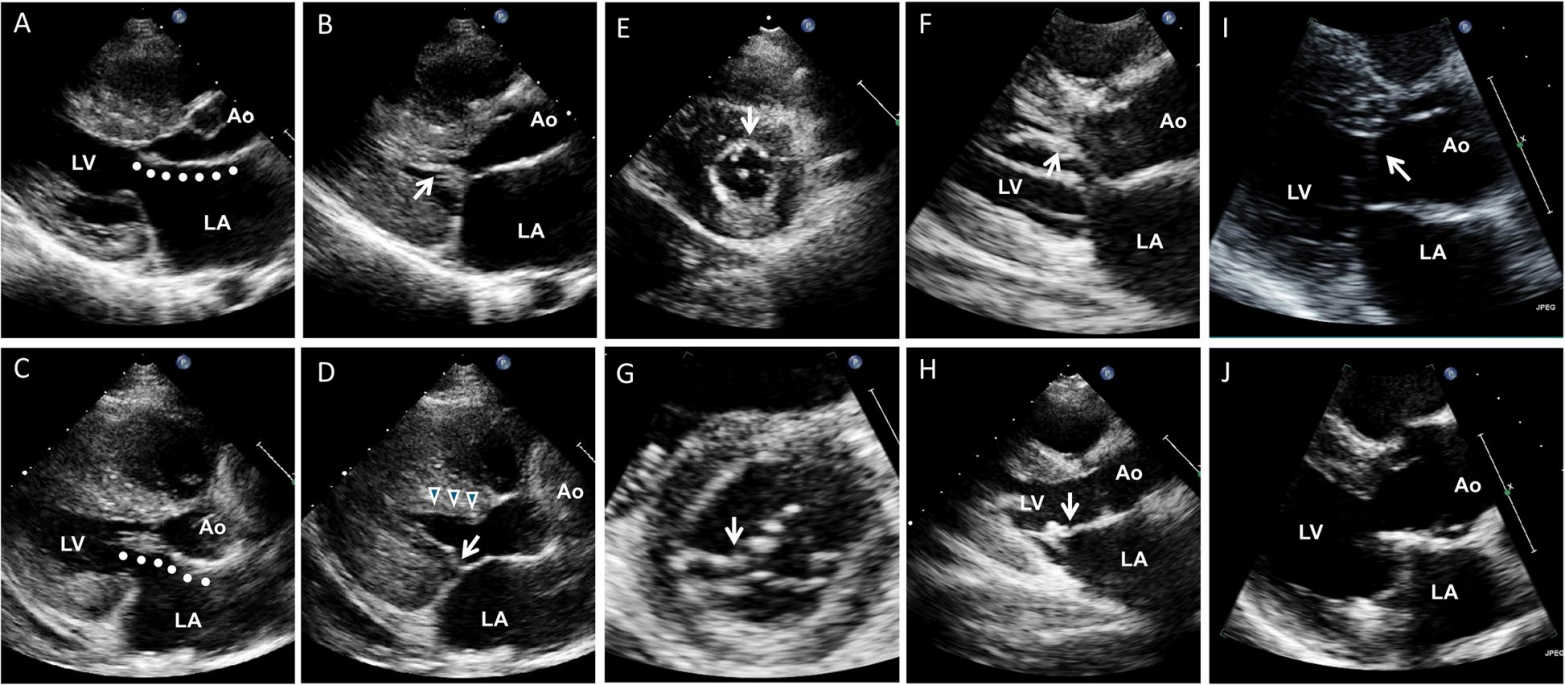
Representative cases of various surgical techniques for elongated mitral leaflets in patients with left ventricular outflow tract obstruction. (**A**, **B**, **C**, **D**) Mitral leaflet plication in addition to myectomy is the most frequently performed technique. (**E**, **F**, **G**, **H**) Edge-to-edge repair or (**I**, **J**) mitral valve replacement is occasionally conducted depending on patient condition. Top and bottom panels show preoperative and postoperative images, respectively

The mitral valve has long been a focus of interest for researchers studying HCM [17]. The initial pathological findings of mitral valve abnormalities in patients with HCM were consistently confirmed through both invasive and noninvasive cardiac imaging studies. The contribution of elongated or enlarged mitral leaflets to the development of SAM-septal contact was highlighted by early researchers using invasive angiography. Pathological and echocardiographic assessments documented elongation of mitral leaflets (Fig. 4) [18]. Elongated leaflets extend or protrude into the LV cavity well above the plane of the mitral annulus, which is important in SAM physiology because the residual protruding portion of the mitral leaflet is not constrained by the pressure difference between the LV and the left atrium, allowing free movement with LV flow, even at low velocities (Fig. 2). Consequently, in addition to myectomy, various surgical techniques aimed at increased surface area or elongated mitral leaflet have been clinically introduced, including mitral leaflet plication (Videos 2, 3) [19–21], edge-to-edge repair (Videos 4, 5) [22, 23], and mitral valve replacement (Videos 6, 7), depending on the specific clinical conditions (Fig. 5).

The challenging issue is whether mitral leaflet elongation is a primary morphological abnormality of HCM. A detailed CMR study revealed that mitral valve leaflets are elongated independently of LV hypertrophy or other disease variables, suggesting a primary phenotypic expression of this heterogeneous disease [24]. The length of the mitral leaflets showed a poor correlation with LV wall thickness, and patients with genotype-positive preclinical HCM showed significantly longer anterior mitral leaflets compared with controls. These findings suggest that fundamental molecular pathways, in addition to a disease-causing sarcomere mutation, play a role in the development of HCM.

**Fig. 6:**
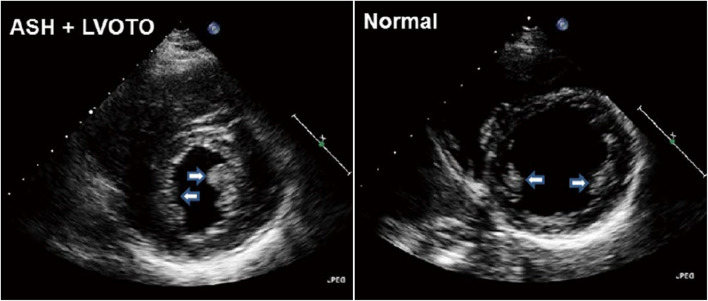
Representative parasternal short axis views of two-dimensional echocardiography demonstrating different papillary muscle positions in hypertrophic cardiomyopathy. Compared with controls, papillary muscles in hypertrophic cardiomyopathy frequently showed a more anteriorly displaced position

Other interesting observational findings include the anterior displacement of the papillary muscle, which results in an anterior position of the coaptation plane of the mitral valve within the LV cavity (Fig. 6) [25, 26]. Anterior and basal displacement of the base of the anterolateral papillary muscle, along with a higher frequency of bifid papillary muscles, is likely the most common pathogenic abnormality in HCM, resulting in closer proximity of the superior papillary muscle and the septum in patients with LVOTO due to SAM [26, 27].

### Anomalous insertion of the papillary muscle directly into the anterior mitral leaflet

**Fig. 7:**
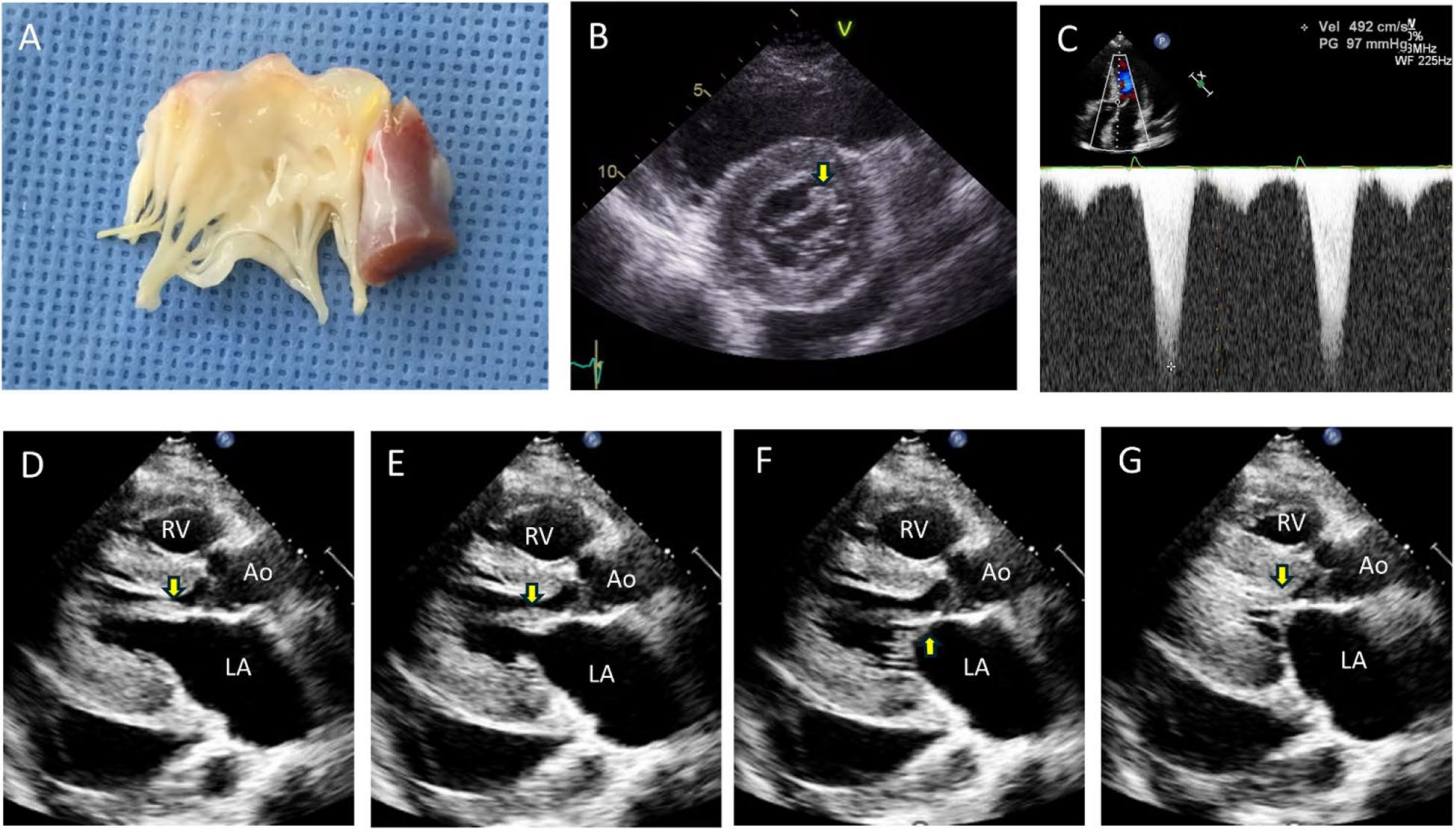
Representative pathologic and echocardiographic images in a patient with hypertrophic cardiomyopathy and anomalous insertion of the papillary muscle directly into the mitral leaflet. (**A**) A pathologic specimen shows a thick papillary muscle attached to the mitral leaflet without chordae tendineae. **B** At the parasternal short axis view, the thick papillary muscle of the anterior leaflet (arrow) is characteristic. **C** Doppler tracing shows a typical delayed peak systolic jet suggestive of left ventricular outflow tract obstruction. (**D**–**G**) With careful tilting of the transducer, direct insertion of the thick papillary muscle directly into the anterior leaflet is demonstrated (arrows)

In a study of 78 mitral valve specimens removed during surgery or autopsy from patients with obstructive HCM, 10 (13%) exhibited a distinctive morphological appearance in which one or both LV papillary muscles were inserted directly into the anterior mitral leaflet [28]. This pathology was subsequently identified using noninvasive imaging modalities, such as CMR [29]. Echocardiography is also useful for preoperative diagnosis of this interesting pathology, showing direct insertion of the papillary muscle into the anterior mitral leaflet without chordae tendineae (Fig. 7A). The anterolateral papillary muscle is more frequently associated with this condition, and the parasternal short-axis view is critical for demonstrating a thickened muscle bundle that crosses just above the anterior mitral leaflet (Fig. 7B, Video 8). As Doppler tracing of the LVOT systolic jet velocity typically exhibits a dagger-shaped profile with a delayed peak, differentiating between SAM and anomalous insertion of the papillary muscle directly into the anterior mitral leaflet—based solely on Doppler tracing without supplementary two-dimensional imaging—is not feasible (Fig. 7C). Therefore, careful evaluation of the subvalvular apparatus is essential, particularly through frame-by-frame analysis (Fig. 7D–G, Video 9). In some cases, off-axis or nonstandard views, achieved by tilting the transducer, are necessary for proper visualization of the subvalvular apparatus. In typical cases, extension of the thick papillary muscle into the anterior mitral leaflet, without thin chordae tendineae, can be easily visualized during the diastolic phase (Fig. 7D, E). During systole, in the absence of SAM with septal contact (Fig. 7F), the thick papillary muscle directly contacts the septum, resulting in the narrowing of the LVOT (Fig. 7G). Preoperative demonstration of this pathology is crucial, as conventional myectomy often fails to alleviate LVOTO with a persistent pressure gradient. In such cases, mitral valve replacement appears to be the procedure of choice for most patients [28].

### Elongated papillary muscle with basal chordal attachment

**Fig. 8:**
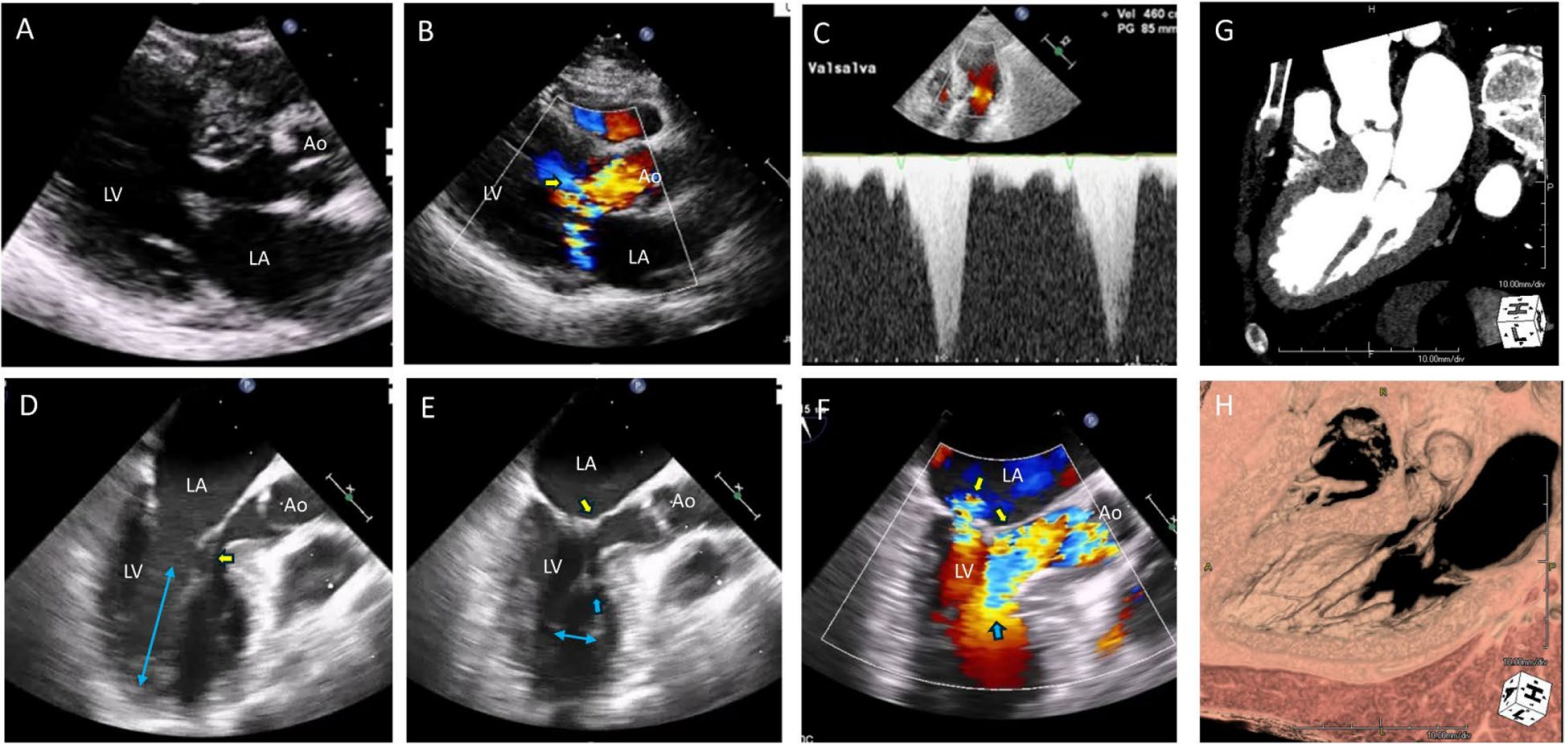
Representative echocardiographic and computed tomographic images showing an elongated papillary muscle causing significant left ventricular outflow tract obstruction without systolic anterior motion of the mitral valve. **A**, **B** Parasternal long-axis views with (**C**) Doppler tracing suggest typical left ventricular outflow tract obstruction. (**D**, **E**) However, transesophageal echocardiographic images clearly show that systolic anterior motion of the mitral valve is absent (yellow arrows): the thick and elongated papillary muscle (D, blue arrows) inserts into the mitral leaflet with thin chordae tendineae extension (D, yellow arrows), which almost contacts the hypertrophied septum during systole, resulting in outflow tract obstruction (E, blue arrow). **G**, **H** Computed tomographic images also demonstrate a thick and elongated papillary muscle inserting to the mitral leaflet with a thin chordae tendineae connection

**Fig. 9:**
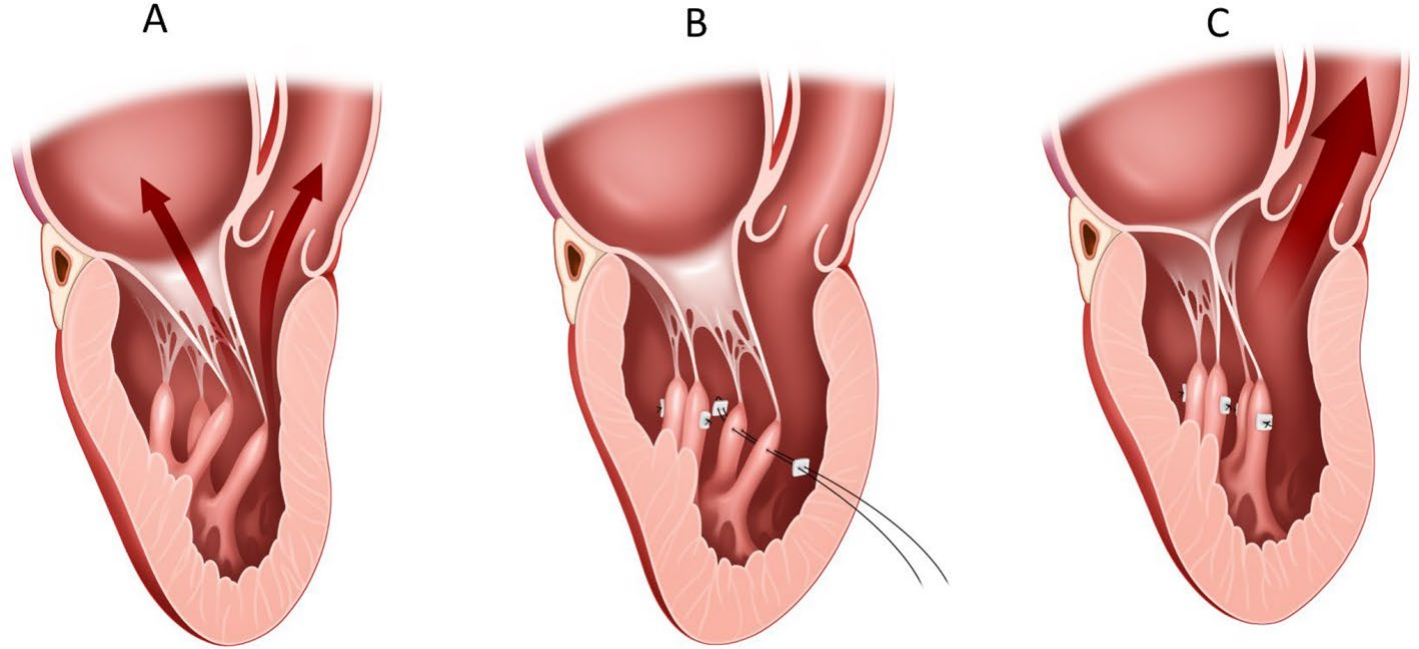
Papillary muscle realignment procedure. **A** Elongated papillary muscles are anteriorly oriented, resulting in left ventricular outflow tract obstruction. **B**, **C** Pledgetted mattress stitches to the adjacent papillary muscle are used to change the orientation of the elongated and mal-oriented papillary muscle. Reproduced from Bryant and Smedira [32], with permission from Elsevier

The orientation of the papillary muscles and their attachment relative to the LVOT, sometimes associated with anomalous papillary muscles, can also be a significant cause of severe LVOTO [30]. This intriguing pathological condition is characterized by abnormally long, hypermobile papillary muscles, which can lead to systolic septal contact and significant LVOTO [27, 30]. As the papillary muscle is attached to the mitral leaflet via thin chordae tendineae, this differs from anomalous papillary muscle insertion. Computed tomography (CT) or transesophageal echocardiography (TEE) with enhanced resolution is typically required for accurate diagnosis of this condition. Flow acceleration in the LVOT, accompanied by characteristic delayed systolic jet tracing, is often observed, suggesting the possibility of classic SAM and septal contact (Fig. 8A–C). TEE can reveal chordal attachment of the papillary muscle (Fig. 8D) and buckling of an elongated and thickened papillary muscle (Fig. 8D, Video 10) during systole, which results in significant flow acceleration in the LVOT without SAM (Fig. 8E, F, Video 11). CT is also valuable for accurate diagnosis of this pathological condition (Fig. 8G, H). Extended septal myectomy for HCM with anomalous mitral papillary muscle or chordae has been proposed as an effective method for relieving LVOTO [31]. This condition can occasionally occur in patients without severe septal hypertrophy, necessitating a specialized papillary muscle realignment procedure (Fig. 9) for complete relief of LVOTO [32]. The primary objective of this procedure is to realign the abnormal orientation of the papillary muscle, which is characterized by anterior displacement that leads to LVOTO. This is achieved by placing a suture at the papillary muscle heads, orienting the papillary muscle posteriorly to prevent septal contact and LVOTO.

**Fig. 10:**
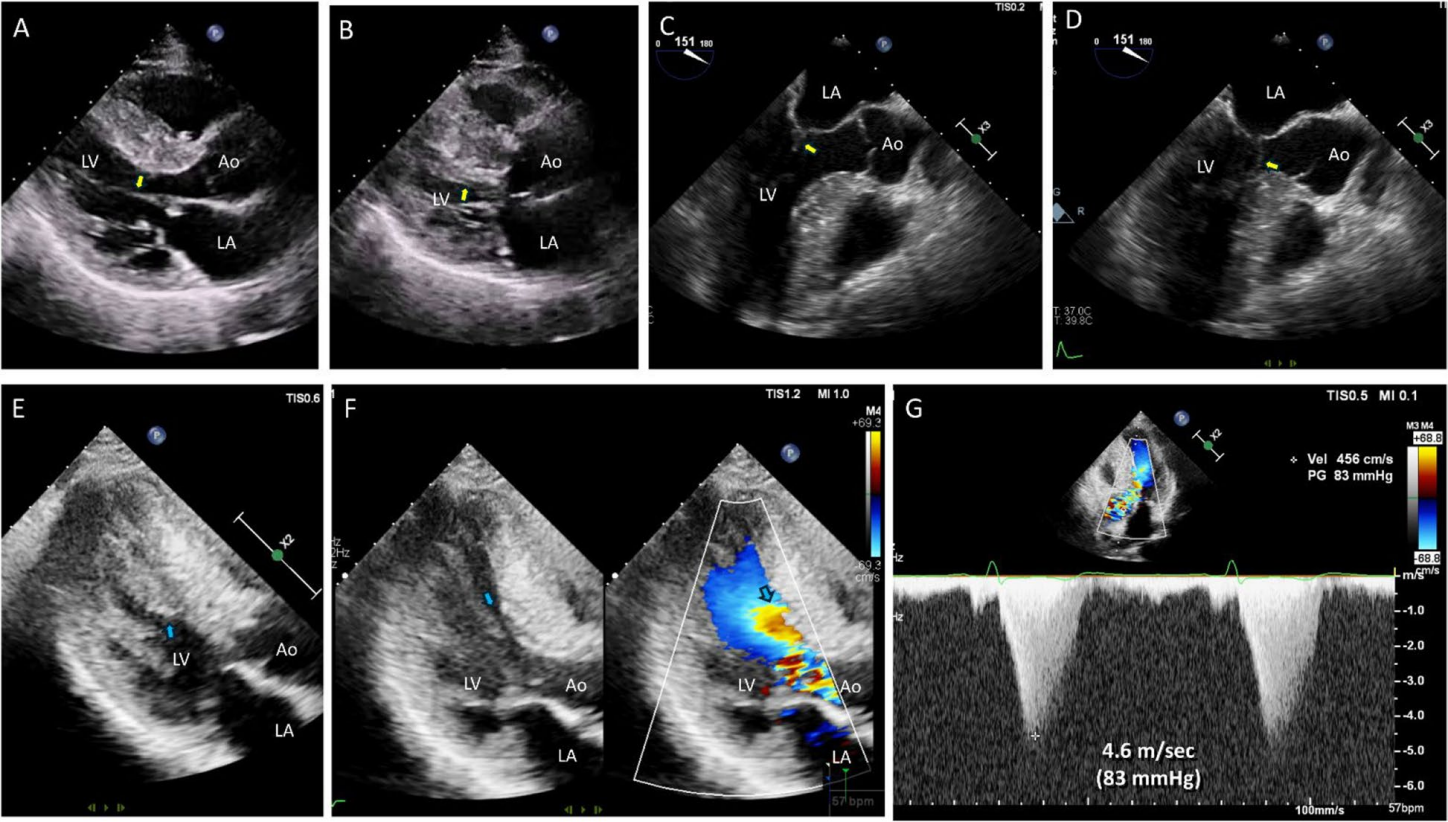
Representative echocardiographic images showing both systolic anterior motion of the mitral valve and elongated papillary muscle causing significant outflow tract obstruction. **A**, **B** In the parasternal long-axis view, a thick and elongated papillary muscle with a thin chordae tendineae insertion that contacts a hypertrophied septum during systole is observed (yellow arrows). **C**, **D** Transesophageal echocardiography shows systolic anterior motion of the mitral valve with septal contact (yellow arrows). **E**, **F** Tilting apical views show anterior movement of the thick and elongated papillary muscle during systole, resulting in flow acceleration (blue arrows). **G** Doppler tracing shows typical dagger-shaped systolic velocity with a delayed peak

**Fig. 11:**
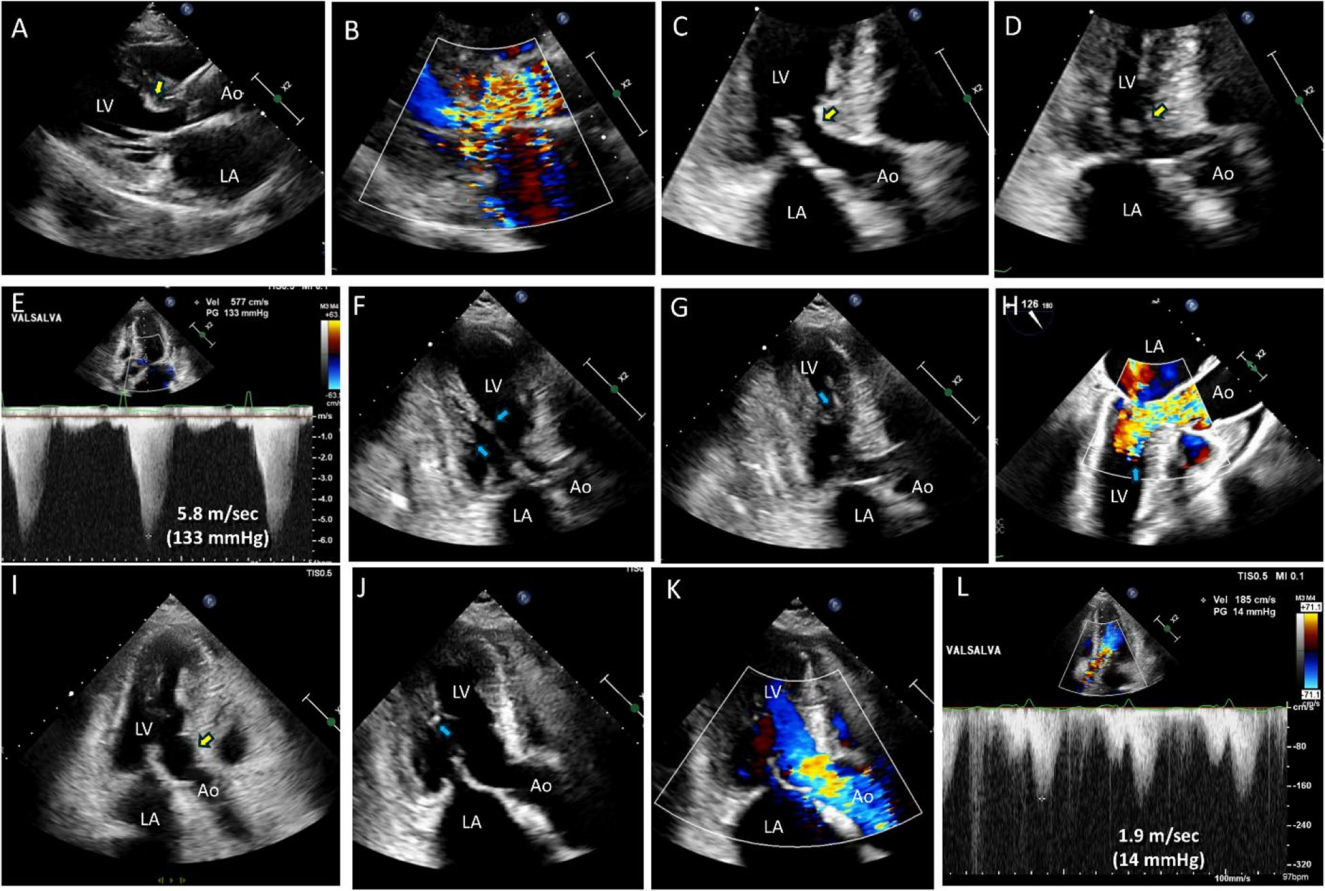
A representative case in which multiple surgical procedures were performed to achieve effective relief of left ventricular outflow tract obstruction. **A** The patient showed a mildly thickened (18 mm) septal wall (yellow arrow) with prominent left ventricular outflow tract obstruction because of (**B**–**E**) prominent systolic anterior motion of the mitral valve and septal contact. Apical views showed (**F**) an anteriorly displaced elongated papillary muscle with thin chordae tendineae insertion to the mitral leaflet, which (**G**, **H**) contacts the septal wall during systole. In addition to (**I**) conventional myectomy, (**J**) using pledgetted mattress stitches, the head of the anteriorly displaced papillary muscle was sutured with the adjacent papillary muscle, which (**K**, **J**) effectively relieved left ventricular outflow tract obstruction

Some patients exhibit both SAM-septal contact and abnormal orientation of the papillary muscles (Fig. 10), which requires multiple surgical interventions, including both myectomy and papillary muscle realignment (Fig. 11, Videos 12–14). The importance of imaging specialists in accurate preoperative diagnoses cannot be overstated.

### Midventricular obstruction

**Fig. 12:**
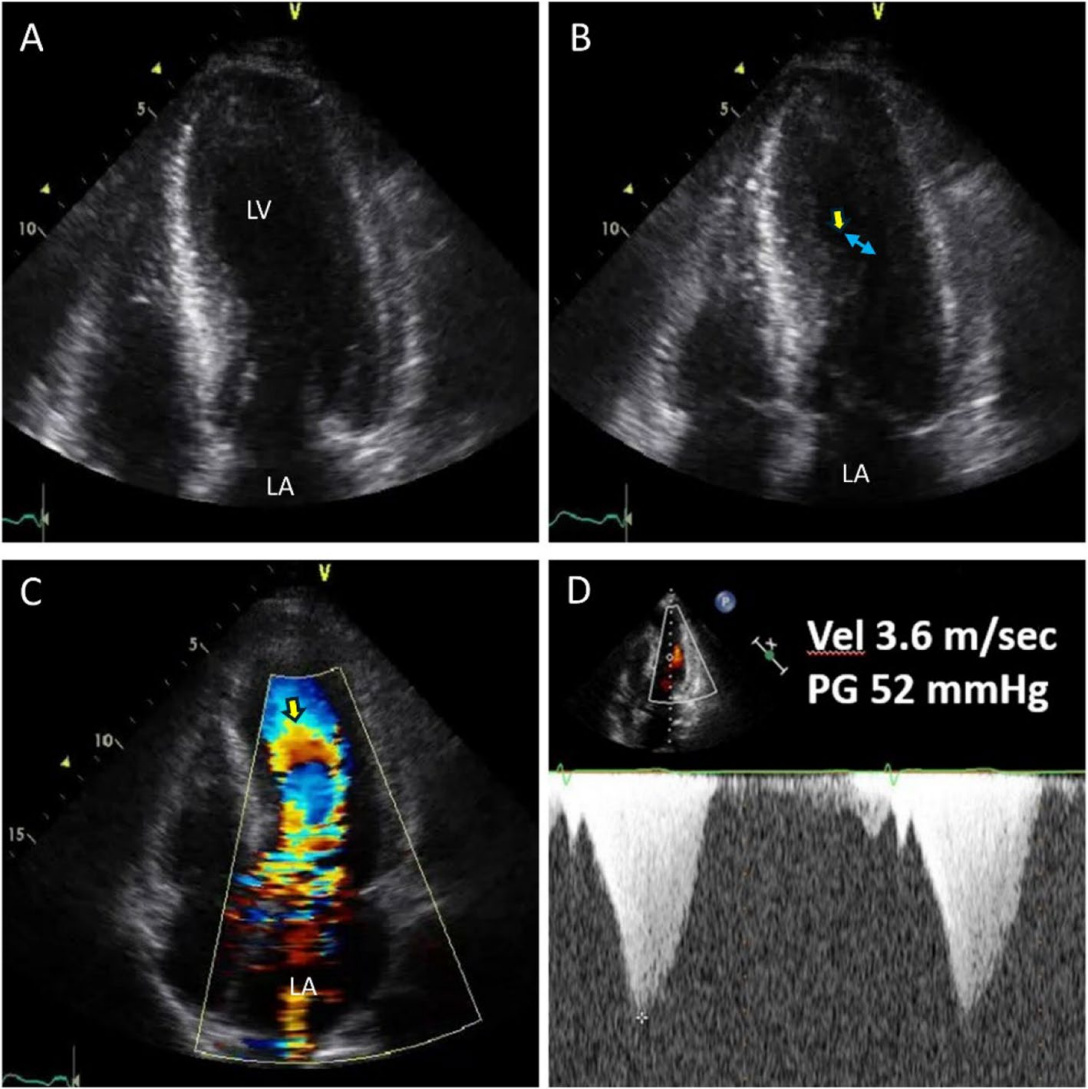
Representative echocardiographic images of midventricular obstruction. During systole, (**A**, **B**) the hypertrophied septum and thick papillary muscle come into contact, which (**C**) induces flow acceleration from the left ventricular midcavity. **D** Doppler tracing shows a typical dagger-shaped increased systolic flow velocity with a delayed peak

**Fig. 13:**
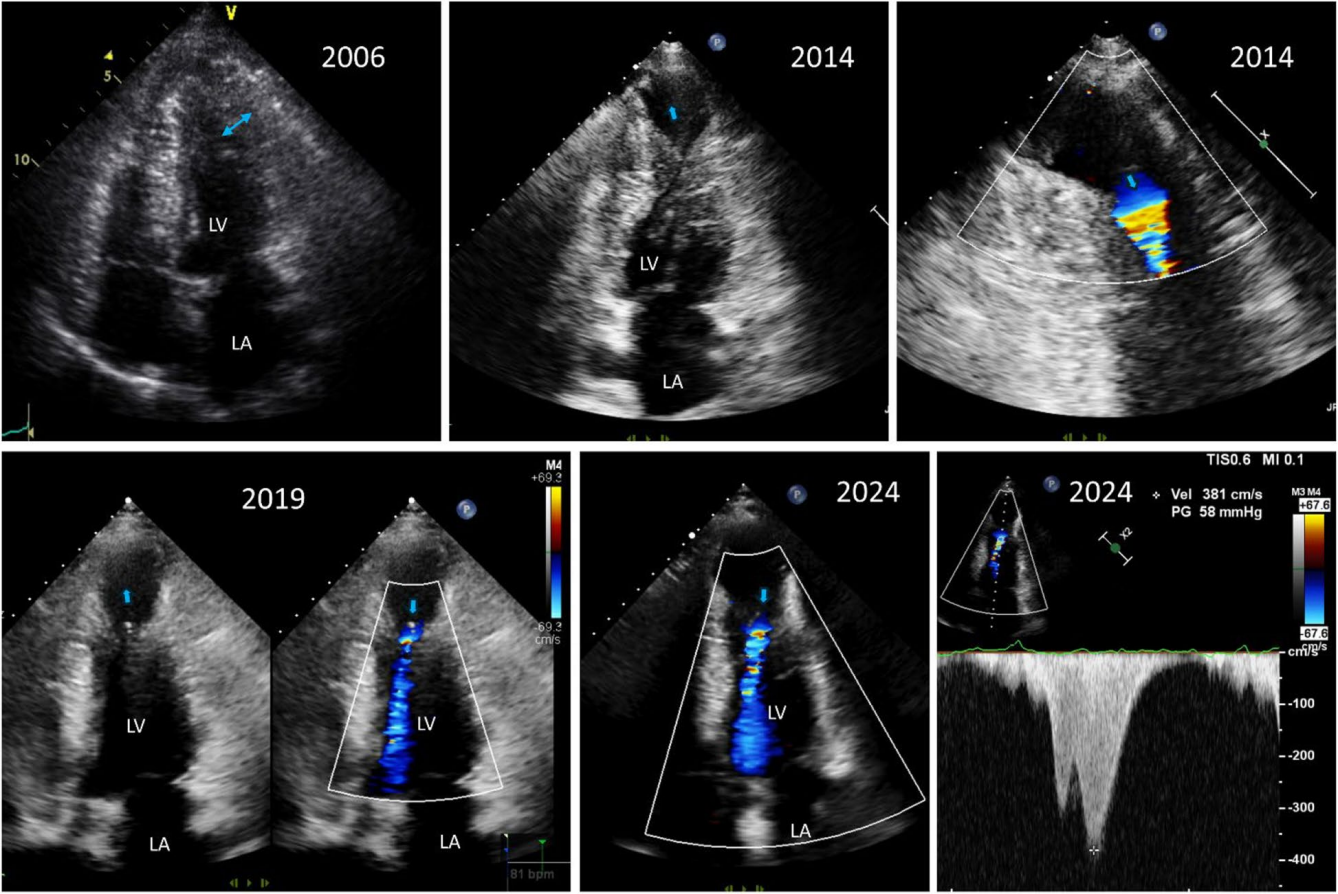
A representative case showing an apical aneurysm and midcavity flow obstruction in a patient with apical hypertrophy. This patient showed typical apical hypertrophy in 2006; during follow-up, apical aneurysm developed. Systolic flow acceleration occurred at the junction between the apical aneurysm and the thick septal and lateral walls

Midventricular obstruction is a less common form of obstruction that occurs deeper within the ventricle. It usually accompanies dynamic ventricular contractility and midsystolic apposition of hypertrophied papillary muscle and the ventricular septum (Fig. 12, Videos 15, 16) [33–36]. In a series of 490 patients with HCM, the prevalence of midventricular obstruction was 9.4%, and it was identified as an independent predictor of adverse outcomes, particularly the combined endpoint of sudden death and potentially lethal arrhythmic events [35]. Notably, continuous wave Doppler tracing cannot differentiate midventricular obstruction from LVOTO due to SAM-septal contact, as the same velocity profile with a typical delayed peak is usually recorded (Fig. 12D). Therefore, careful echo-Doppler evaluation is essential, as underdiagnosis or underestimation of midventricular obstruction has been documented. Notably, the long-term prognosis does not differ between patients with typical LVOTO due to SAM-septal contact and those with midventricular obstruction [35]. Increases in the rate of sudden death in patients with midventricular obstruction and apical aneurysms have been reported, and these events are considered high-risk factors associated with sudden death [34, 37, 38]. Therefore, serial follow-up is necessary to monitor the development of apical aneurysms in this patient population. This condition should be differentiated from apical aneurysm in patients with apical HCM [39, 40], which is not associated with a thick and prominent papillary muscle. In apical HCM, midsystolic flow obstruction is not present before development of apical aneurysm, which typically takes a long time (Fig. 13).

### Challenging issues in diagnosis

The dynamic nature of LVOTO in patients with HCM is well recognized, and maneuvers aimed at decreasing preload or afterload while increasing contractility are essential for diagnosing latent LVOTO (Fig. 3). The Valsalva maneuver and exercise are among the most common provocative techniques in clinical practice. However, in the elderly population, the application of these maneuvers might be limited because of difficulties in following instructions to correctly perform the Valsalva maneuver. A recent study reported that sitting without engaging in strenuous maneuvers can effectively reveal latent LVOTO, particularly in elderly patients [41].

**Fig. 14:**
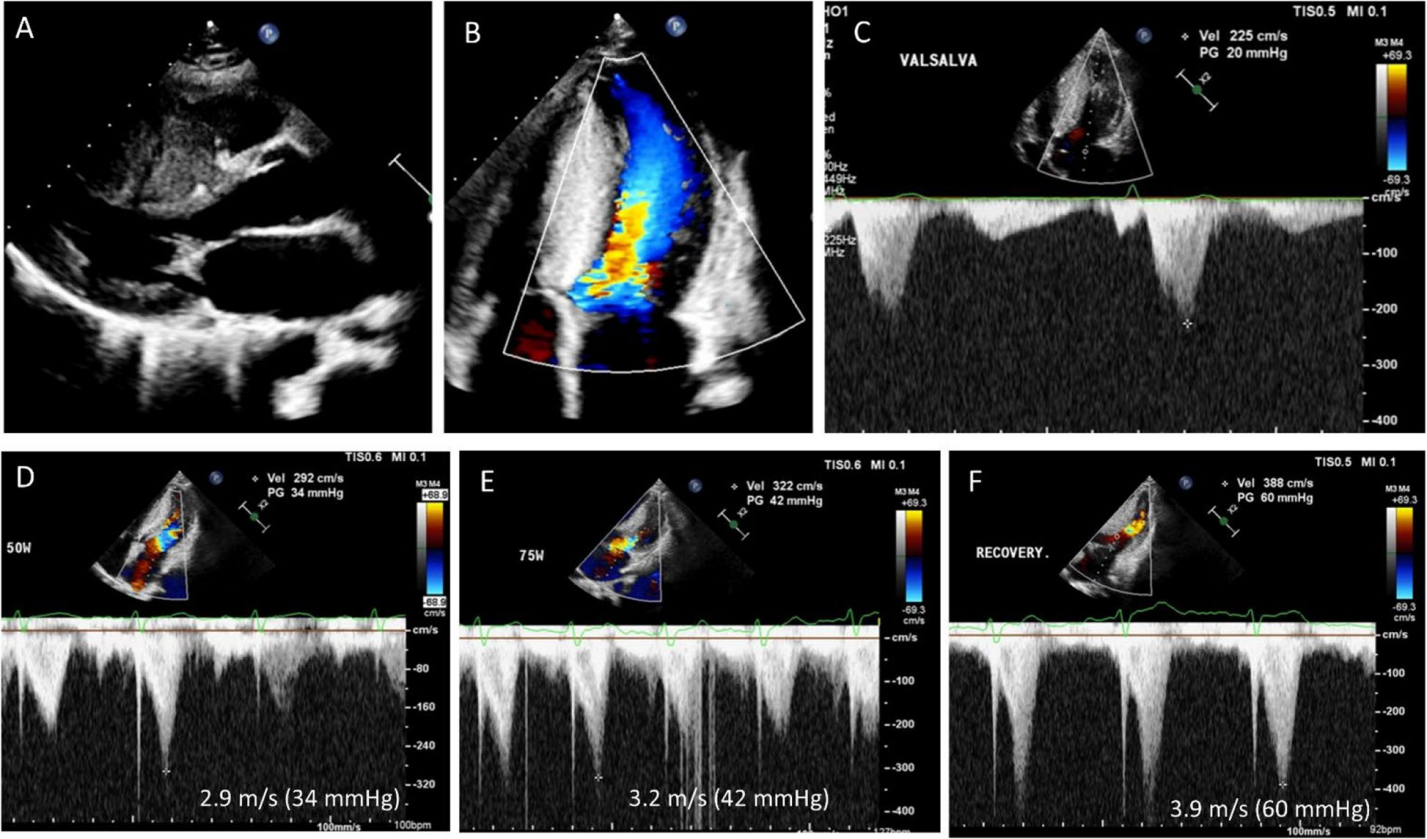
A representative case showing significant left ventricular outflow tract obstruction immediately after exercise. A 32-year-old woman developed repeated episodes of syncope after running short distances. The syncope usually developed after the exercise; during exercise, she did not experience any discomfort. (**A**–**C**) Resting echocardiography showed typical asymmetrical septal hypertrophy without systolic anterior motion of the mitral valve or left ventricular outflow tract obstruction at rest and with the Valsalva maneuver. (**D**, **E**) During a semi-supine bicycle exercise, significant left ventricular outflow tract obstruction was absent; (**F**) however, immediately after exercise, a delayed peak velocity of 3.9 m/sec (60 mmHg) was recorded

Exercise is a more physiological approach for diagnosing latent LVOTO, with several key points that need to be emphasized. The semi-supine bicycle with a tilting position is the preferred method compared with the supine exercise test, as it allows easier recording of Doppler signals without compromising the physiological response to exercise. Another important consideration is the need to carefully record the post-exercise period. In some patients who experienced recurrent syncope immediately after exercise or running, resting echocardiograms with the Valsalva maneuver did not reveal any evidence of significant LVOTO. During exercise testing, LVOTO might not be apparent; however, immediately after exercise, a typical delayed peak systolic velocity can be measured (Fig. 14).

### Management of LVOTO: a significant paradigm shift

The current guidelines recommend medications that target negative inotropic effects, which include β-adrenergic blockers, L-type calcium channel blockers, and disopyramide [9, 10]. While these drugs are widely used, they have not been tested in large, placebo-controlled clinical trials. The consensus is that these medications cause a moderate reduction in the intraventricular pressure gradient and alleviate symptoms in some patients.

While the recommended drugs affect nonspecific blockers at the cell membrane, recent advances in molecular biology have created momentum for targeting a more specific action site related to myocardial contractility. LV hypertrophy in HCM is now understood to be caused by myocardial hypercontractility, which results from an excess of actin-myosin cross-bridges in the sarcomere [42]. Myosin ATPase inhibition has demonstrated a remarkable reduction in this excessive cross-bridging, effectively blocking LVOTO [43, 44]. The U.S. Food and Drug Administration approved these treatments, and both American and European guidelines now include class I and IIa recommendations for their use. These myosin inhibitors are anticipated to modify the disease course of patients with HCM.

**Fig. 15:**
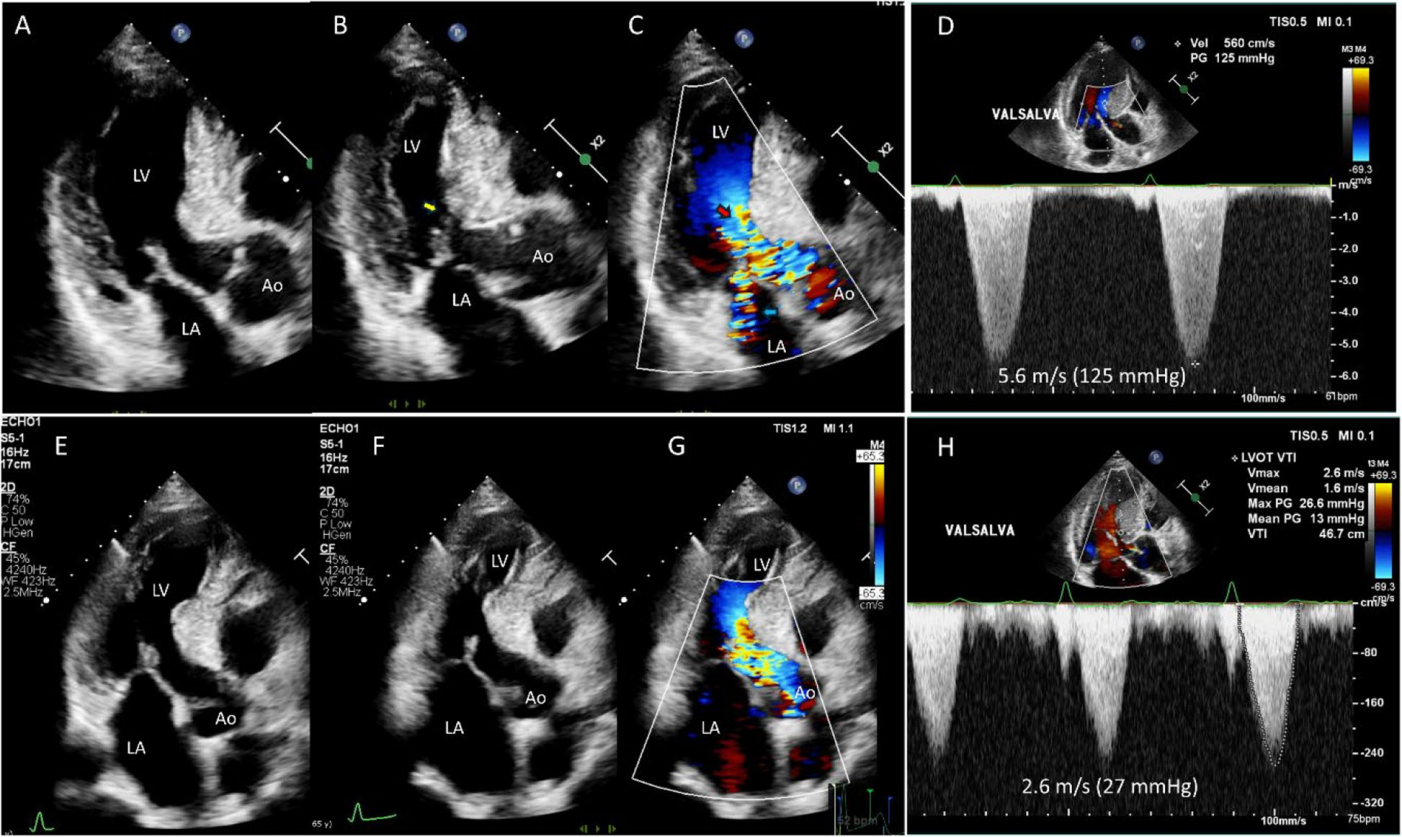
A representative case showing dramatic improvement of left ventricular outflow tract obstruction with administration of a cardiac myosin inhibitor. (**A**–**D**) A 56-year-old man with prominent systolic anterior motion of the mitral valve (SAM) and significant left ventricular outflow tract (LVOT) obstruction experienced intractable dyspnea with conventional medications including β-receptor blockers. With the addition of a cardiac myosin inhibitor, (**E**, **F**) SAM became less prominent with loss of septal contact, which resulted in (**G**, **H**) markedly decreased flow acceleration in LVOT and resolution of mitral regurgitation

**Fig. 16:**
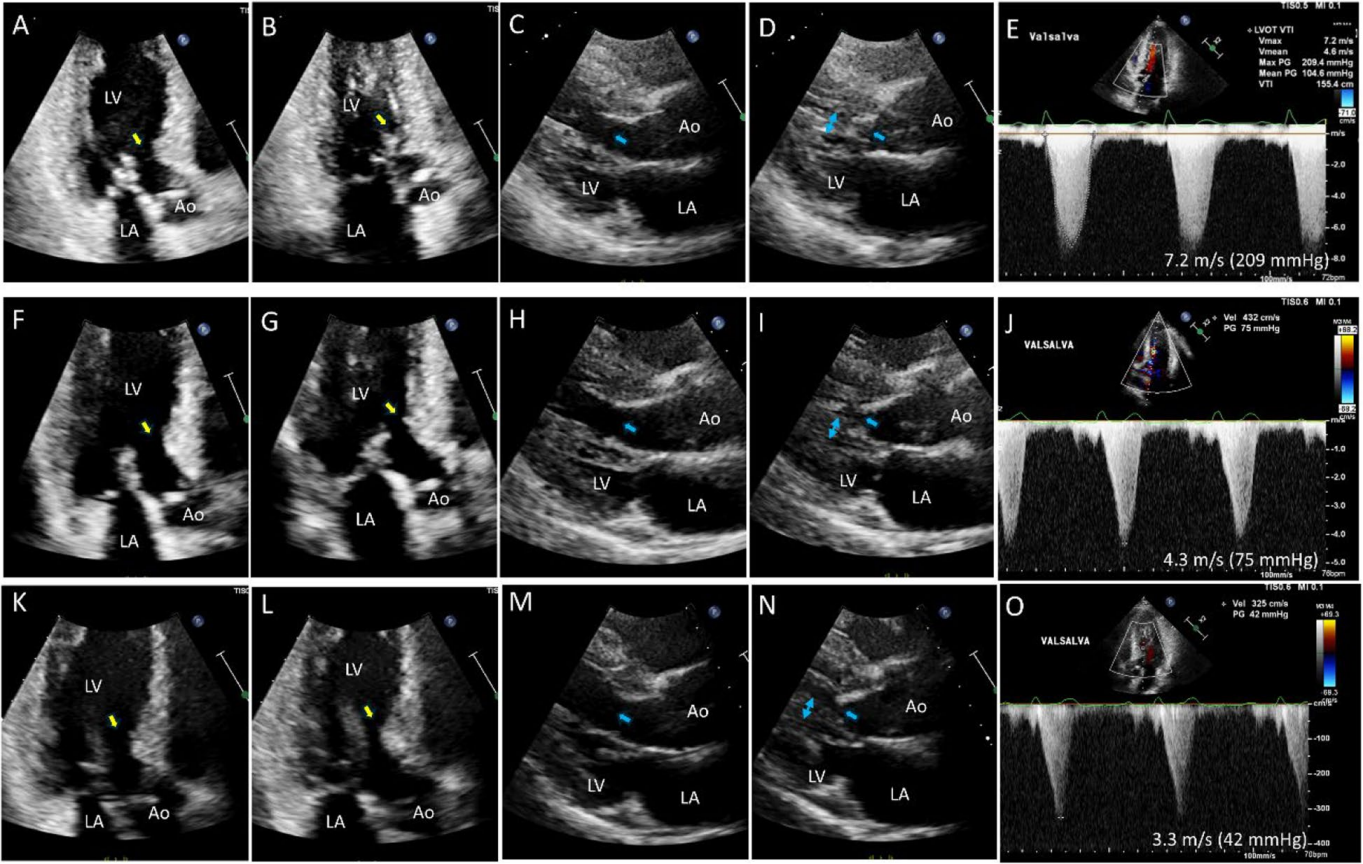
A representative case showing improvement of multiple mechanisms of left ventricular outflow tract obstruction with administration of a cardiac myosin inhibitor. (**A**, **B**) A 67-year-old man complained of severe effort dyspnea after a successful percutaneous coronary intervention. Echocardiography showed prominent systolic anterior motion of the mitral valve (SAM) with septal contact. (**C**, **D**) Elongated papillary muscle also contributed to (**E**) left ventricular outflow tract (LVOT) obstruction. At 1 month of administration of a cardiac myosin inhibitor, (**F**, **G**) SAM-septal contact was lost, but (**H**, **I**) septal contact of the elongated papillary muscle persisted, and (**J**) the peak velocity of LVOT flow decreased significantly. Three months after medication, (**K**–**N**) both SAM and elongated papillary muscle-septal contact were absent, which resulted in (**O**) marked decrease in LVOT peak velocity

Real-world experience with cardiac myosin inhibitors is quite remarkable. Within a short treatment period less than 1 month, dramatic symptomatic improvements, including a significant reduction in SAM-septal contact, are frequently observed (Fig. 15, Videos 17–19). An especially noteworthy aspect of this medication is its effectiveness in alleviating LVOTO through various mechanisms. In addition to the initial relief of SAM-septal contact achieved with the medication (Fig. 16F–J, Videos 20–22), ongoing treatment can lead to a successful reduction in LVOTO caused by elongation of the papillary muscle (Fig. 16K–O, Video 23).

## LVOTO associated with various disease entities

### Sigmoid septum in the elderly population

**Fig. 17:**
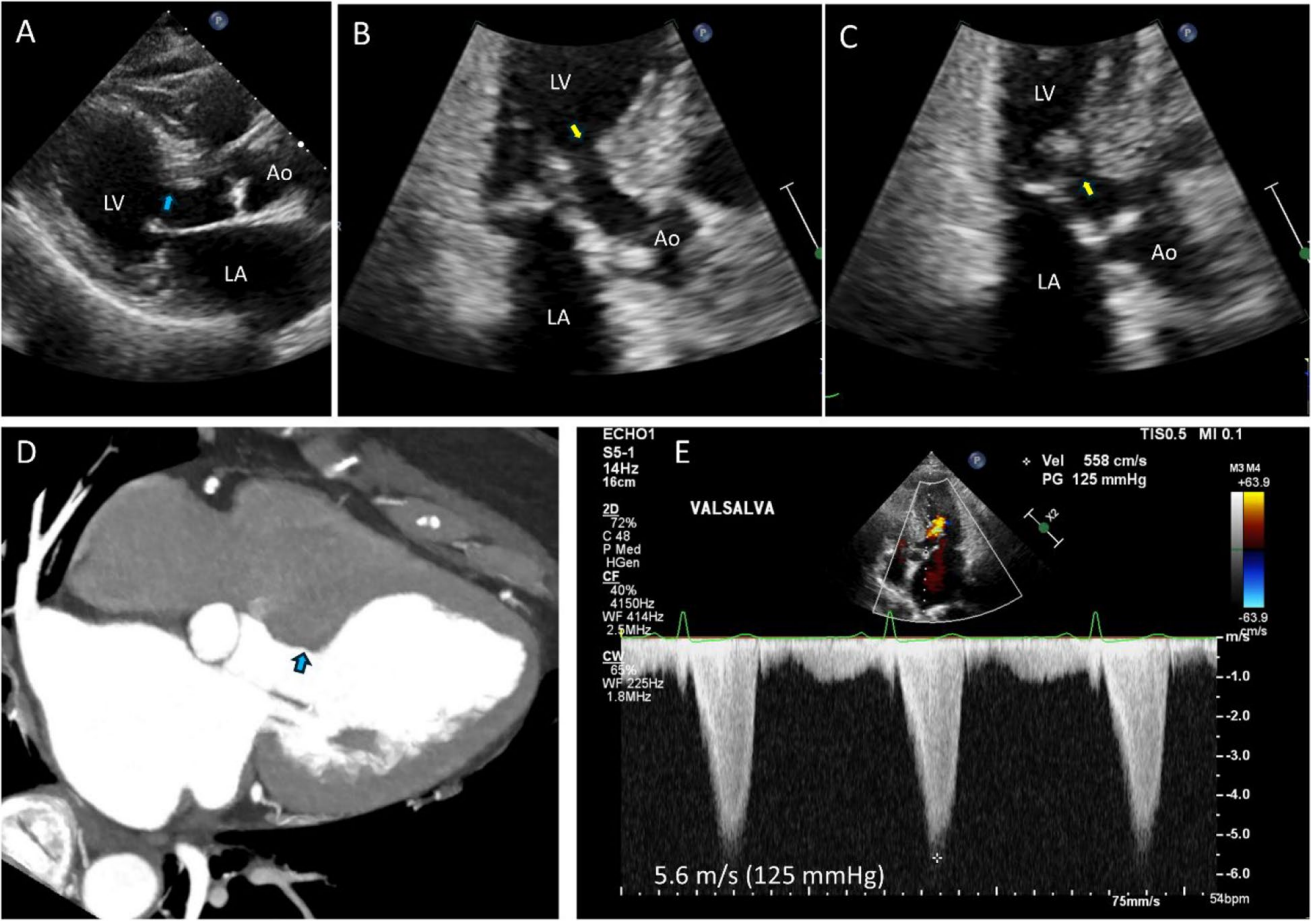
Representative echocardiographic and computed tomographic images of the sigmoid septum. (**A**, **D**) Parasternal long-axis view showing discrete septal thickening with characteristic angulation between the ventricular septum and aorta (blue arrows). (**B**, **C**) Prominent systolic anterior motion of the mitral valve contacts the septum, resulting in (E) elevated left ventricular outflow tract flow velocity and obstruction

**Fig. 18:**
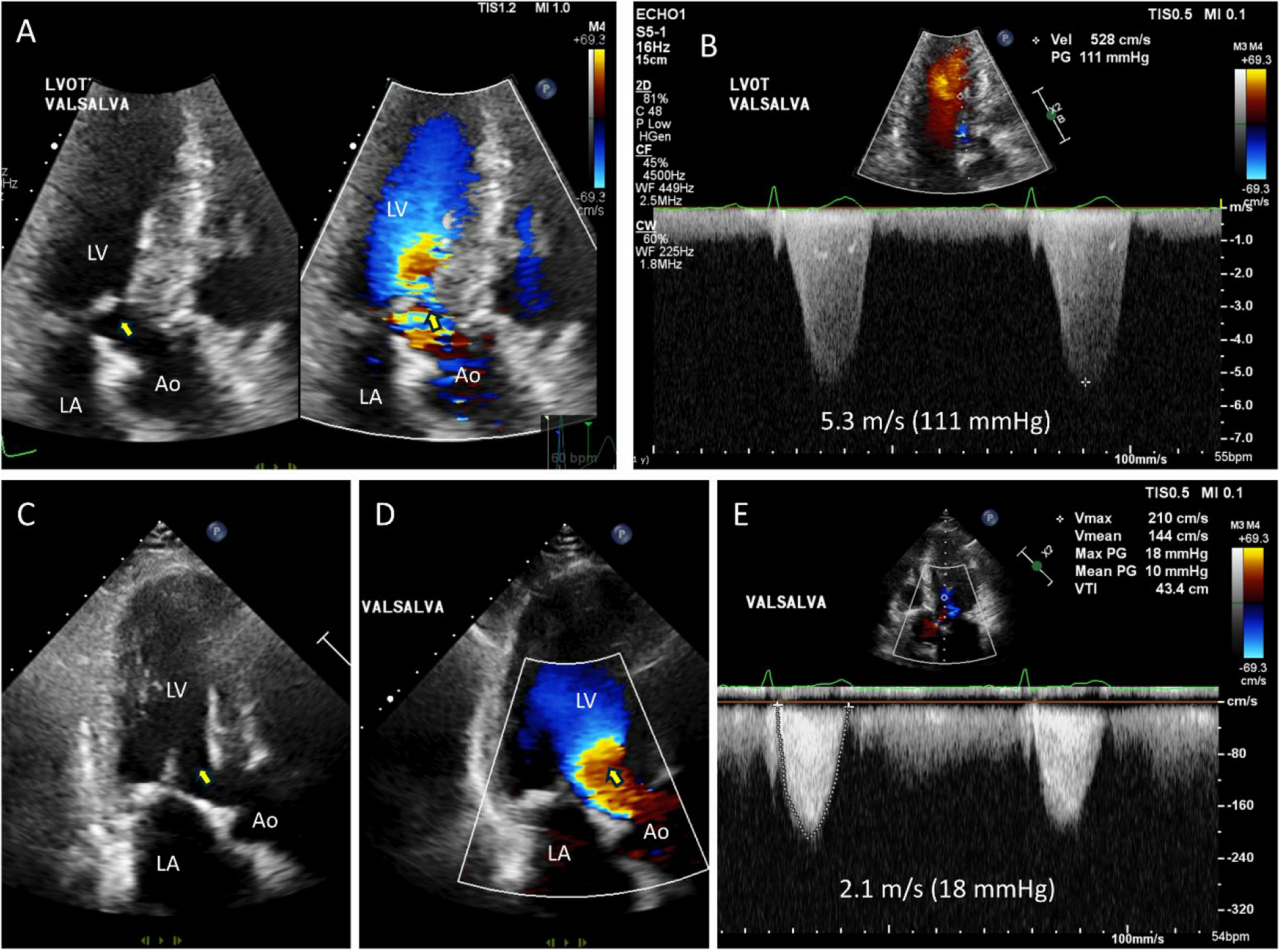
A representative case showing the effect of a cardiac myosin inhibitor in a sigmoid septum with significant left ventricular outflow tract (LVOT) obstruction. An 82-year-old man experienced intractable exertional dyspnea despite multiple medications including β-receptor blocker. Echocardiography showed (**A**) sigmoid septum and prominent SAM-septal contact resulting in (**B**) very high peak velocity at the LVOT. (**C**, **D**) Loss of SAM-septal contact occurred with additional treatment with a cardiac myosin inhibitor, resulting in (**E**) effective relief of LVOT obstruction

The sigmoid septum is an age-related change characterized by a decrease in the angle between the ventricular septum and the aorta. This change occurs as a result of shortening of the LV axis and increased tortuosity of the aorta accompanied by dilation [45]. It is marked by a sigmoid-shaped septum with localized or discrete upper septal hypertrophy (Fig. 17A, D). Prominent SAM and its contact with the septum can lead to significant LVOTO, resulting in marked symptomatic deterioration (Fig. 17B, C, E) [46, 47]. The recently introduced cardiac myosin inhibitors have proven effective in alleviating the SAM-septal contact and LVOTO associated with the sigmoid septum, conditions that are not easily managed by conventional β-receptor blockers or calcium channel blockers (Fig. 18, Videos 24–26). Given that surgical myectomy carries relatively high risks and its efficacy has not been established in elderly patients with a sigmoid septum, the availability of effective medical therapeutic options is encouraging.

### Aortic stenosis

**Fig. 19:**
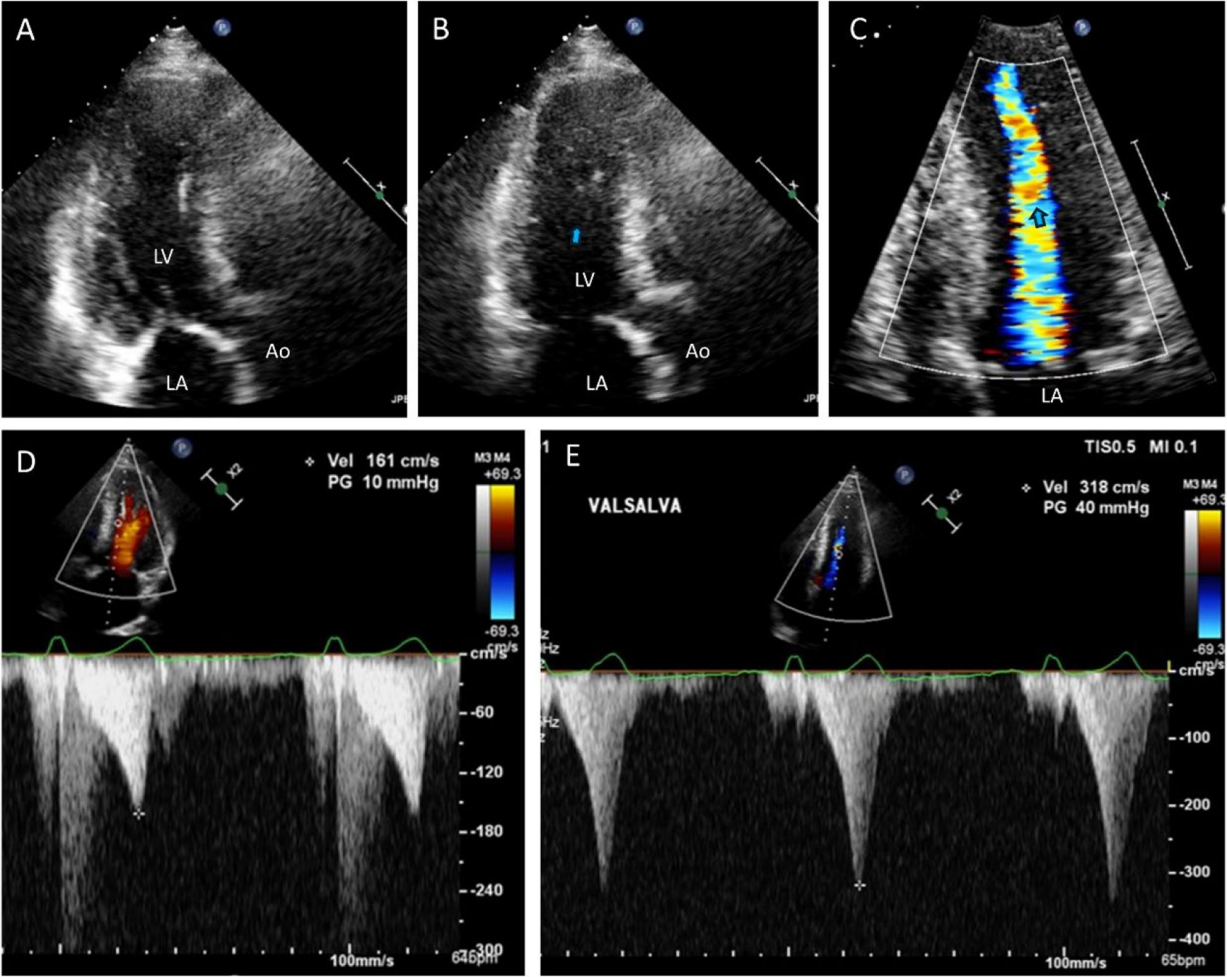
A representative case showing systolic intracavitary gradients after successful surgical aortic valve replacement. After successful aortic valve replacement, apical views show (**A**, **B**) strong contact between the hypertrophied papillary muscle and ventricular septal wall with (**C**) marked flow acceleration. Doppler tracing shows (**D**) typical dagger-shaped peak systolic velocity, which (**E**) intensifies with the Valsalva maneuver

An intriguing Doppler tracing exhibiting typical delayed peak, which suggests systolic intracavitary gradients following aortic valve replacement, was reported more than 30 years ago [48]. In a later study, the incidence of this phenomenon after surgical aortic valve replacement (SAVR) was estimated to be 14% [49]. Both studies noted the rarity of SAM, indicating different underlying mechanisms, including cavitary obliteration or midventricular obstruction. A small, hyperdynamic, and asymmetrically hyperdynamic LV was identified as a risk factor for abnormal systolic intraventricular flow velocities after SAVR. High afterload resulting from aortic stenosis leads to compensatory LV hypertrophy and chronic pressure overload. Following successful SAVR, a rapid decrease in afterload can enhance LV contractility from the chronically hypertrophied LV, potentially resulting in cavity obliteration (Fig. 19, Videos 27, 28). As transcatheter aortic valve implantation (TAVI) has also been shown to reduce LV afterload, this intracavitary pressure gradient can be readily anticipated. Although the initial interpretation indicated that this intriguing Doppler tracing appeared benign, instances of sudden hemodynamic deterioration following successful intervention for aortic stenosis have been documented, leading to the term “suicide LV” to describe this potentially fatal event [50, 51]. The almost immediate alleviation of the transvalvular gradient after SAVR or TAVI exposes the LV to a sudden and significant reduction in afterload. Consequently, LV hypercontractility and dynamic intraventricular gradients can develop or worsen, resulting in a potential resistant circulatory collapse [52, 53]. More importantly, a systematic review of clinical case reports reported that LVOTO due to prominent SAM-septal contact, rather than midventricular obstruction, is a more common cause of “suicide ventricle” [54].

**Fig. 20:**
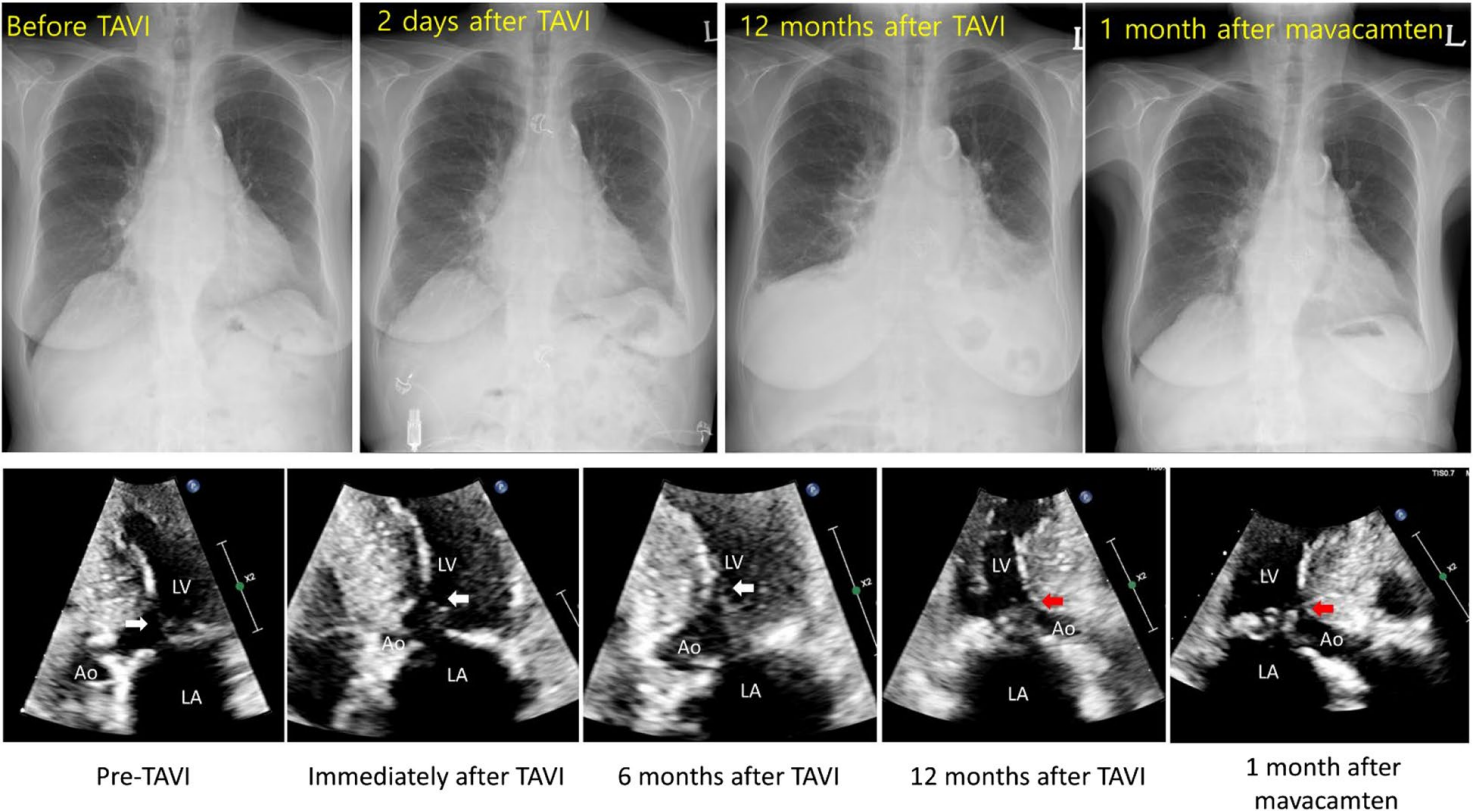
A representative case showing the development of progressive heart failure after uneventful transcatheter aortic valve implantation. An 87-year-old woman developed progressive dyspnea 12 months after uneventful transcatheter aortic valve implantation. **A** Chest x-ray showed pulmonary edema and bilateral pleural effusion. **B** Echocardiography showed progressive development of prominent systolic anterior motion of the mitral valve resulting in septal contact (arrows). A cardiac myosin inhibitor dramatically decreased SAM-septal contact, with improvement of symptoms and chest x-ray findings. Reproduced from Jo et al. [55], available under the Creative Commons license

It appears that SAM-septal contact following SAVR or TAVI can present with various clinical manifestations, potentially leading to progressive heart failure rather than a “suicide ventricle.” In a recent case report, an 87-year-old woman developed progressive heart failure after an uneventful TAVI procedure. Echocardiography clearly demonstrated the emergence of significant SAM-septal contact post-TAVI, which did not improve with administration of a β-receptor blocker (Fig. 20) [55]. Mavacamten dramatically reduced SAM-septal contact, and the patient’s condition progressively improved with alleviation of LVOTO. Thus, a cardiac myosin inhibitor effectively relieved LVOTO caused by SAM, regardless of the underlying physiological mechanisms of LV hypertrophy, whether due to increased afterload in aortic stenosis or hypertrophy without LV pressure overload in HCM. ASH is reported to occur in approximately 10% of patients with hemodynamically significant valvular aortic stenosis [56]. Further investigations are necessary to clarify the mechanisms behind the development of SAM and significant LVOTO in patients experiencing typical pressure overload.

### Takotsubo syndrome

**Fig. 21:**
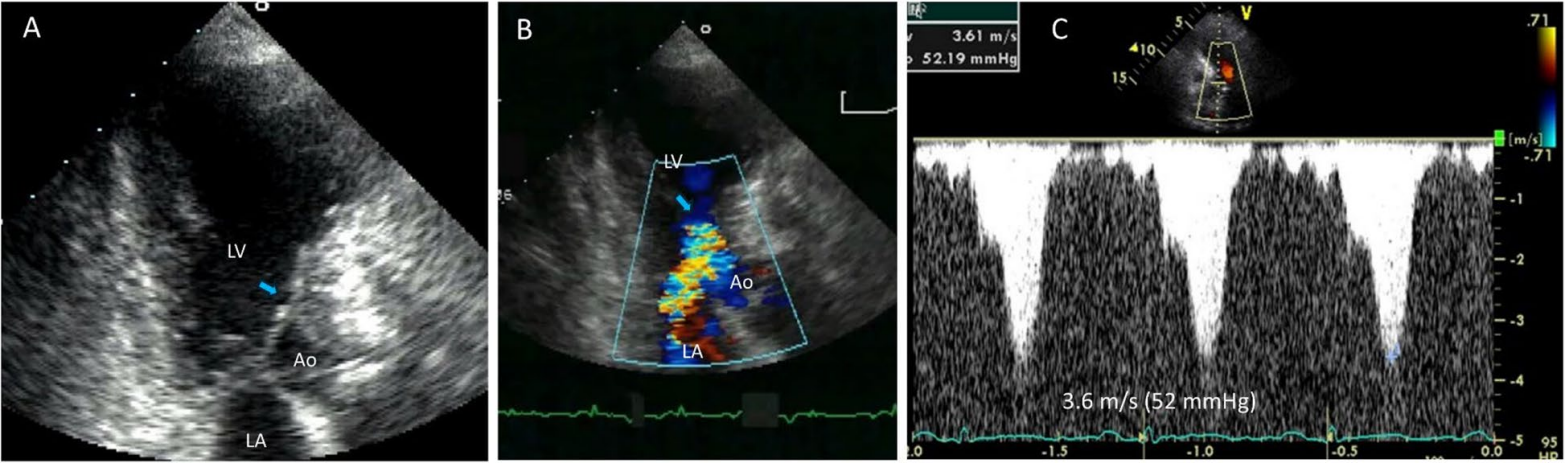
A representative case showing left ventricular outflow tract obstruction associated with Takotsubo syndrome. A 68-year-old woman developed severe dyspnea after uneventful bronchoscopy. **A** Echocardiography showed typical apical ballooning and prominent systolic contact of the mitral valve and ventricular septum (arrow) resulting in (**B**, **C**) marked flow acceleration and left ventricular outflow tract obstruction

LVOTO has been reported in patients with Takotsubo syndrome, particularly those exhibiting typical apical ballooning (Fig. 21, Video 29). A recent study from a multicenter registry of patients with Takotsubo syndrome found that 58 of 322 patients (18%) experienced LVOTO [57]. Cardiogenic shock, which is associated with a more severe in-hospital course, was common. While the long-term outcomes appear to be similar to those of patients without LVOTO, the clinical significance of early recognition of this potential complication in patients with Takotsubo syndrome and typical apical ballooning supports the need for vigilance of attending physicians.

### Mitral annular calcification

**Fig. 22:**
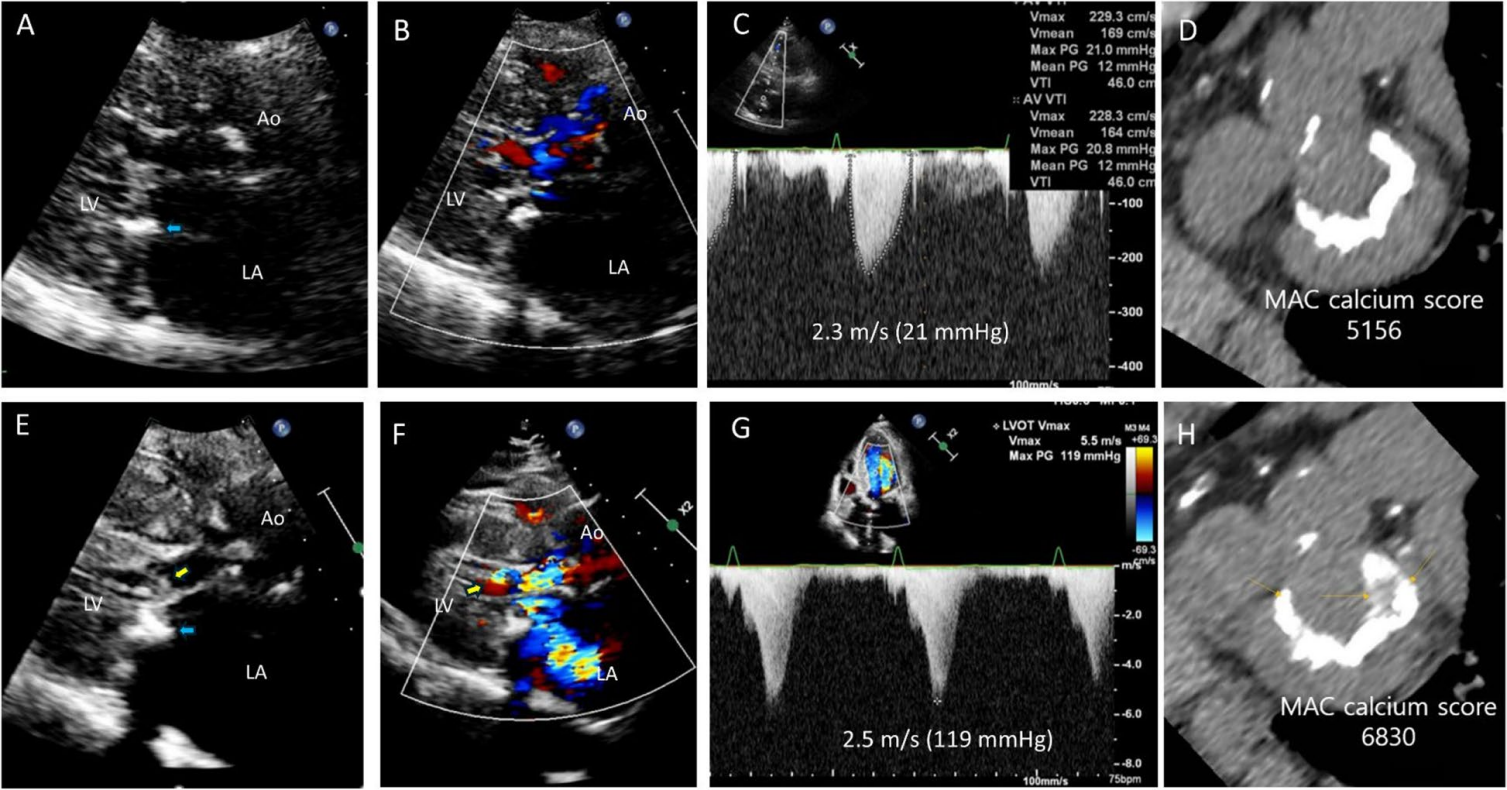
A representative case showing the contribution of progression of mitral annular calcification to left ventricular outflow tract obstruction. A 67-year-old woman with no history of hypertrophic cardiomyopathy showed (**A**, **B**) mitral annular calcification and hypertrophied papillary muscle without (**C**) significant left ventricular outflow tract (LVOT) obstruction. **E**–**G** Four years later, severe flow acceleration and LVOT obstruction were documented. **D**, **H** Computed tomography clearly showed the progression of mitral annular calcification with increased calcification extent and calcium score

Mitral annular calcification (MAC) is frequently observed in patients with symptomatic HCM [58, 59]. In a surgical series study, MAC was associated with a higher rate of mitral valve replacement compared with the absence of MAC [59]. One significant finding was that MAC leads to incremental anterior displacement of the mitral valve into the LVOT, which might contribute to the development of SAM-septal contact [58]. With the marked increase in the elderly population—characterized by a higher prevalence of MAC, its progression, progressive tortuosity of the aorta, and an increasing prevalence of sigmoid septum—there will be a significant increase in elderly patients experiencing SAM-septal contact that results in hemodynamically significant LVOTO without HCM (Fig. 22, Videos 30, 31). Notably, the association between MAC progression and SAM-septal contact, along with the progressive anterior displacement of the mitral valve, suggests that preventing MAC progression could be a rational strategy for mitigating the development of LVOTO in the elderly population.

## Conclusions

Over the past several decades, the understanding of LVOTO in patients with HCM has dramatically increased. LVOTO was initially identified through invasive cardiac catheterization and considered a pathognomonic finding of HCM. Noninvasive imaging modalities, particularly the echo-Doppler technique, have significantly enhanced the understanding of the mechanisms of LVOTO. Further investigations showed that, while LV hypertrophy is the most prominent pathological finding of HCM, alterations in the mitral valve and subvalvular structures can develop independently and significantly contribute to the development of LVOTO in HCM. Therefore, in addition to the classic myectomy aimed at LV hypertrophy, various surgical techniques have been developed to manage these additional abnormalities involving the mitral valve and the subvalvular structures. The role of imaging specialists in providing insights into the specific mechanisms of LVOTO cannot be overstated, and further advances in the precise evaluation of the mitral valve and subvalvular apparatus—including the location and orientation of papillary muscles and their chordae connections— are essential.

A significant advancement in this field is the successful clinical introduction of a more targeted medical treatment aimed at addressing the exaggerated actin-myosin cross-bridging, a key pathophysiological mechanism of HCM. The treatment and management of patients with HCM and LVOTO is expected to change dramatically with success of cardiac myosin inhibitors. Notably, LVOTO is not limited to patients with HCM and is expected to be encountered more frequently in our aging society. Conditions such as sigmoid septum, degenerative aortic stenosis, and MAC are increasingly associated with significant LVOTO in the elderly population. The effective relief of LVOTO using cardiac myosin inhibitors in non-HCM patients indicates a common pathophysiological link between LV hypertrophy in HCM and that resulting from typical pressure overload. Further clinical investigations are essential to improve the clinical outcomes of such patients.

## Supplementary Information

Supplementary Material 1. A magnified three-chamber view showing prominent systolic motion of the mitral valve and septal contact that results in left ventricular outflow tract obstruction.

Supplementary Material 2. A parasternal long-axis view before myectomy and plication of the anterior mitral leaflet.

Supplementary Material 3. A parasternal long-axis view after myectomy and plication of the anterior mitral leaflet.

Supplementary Material 4. A parasternal short-axis view before edge-to-edge repair of the mitral leaflet.

Supplementary Material 5. A parasternal short-axis view after edge-to-edge repair of the mitral leaflet.

Supplementary Material 6. A magnified parasternal long-axis view before mitral valve replacement.

Supplementary Material 7. A magnified parasternal long-axis view after mitral valve replacement.

Supplementary Material 8. A characteristic parasternal short-axis view showing the prominently thick papillary muscle located just above the anterior mitral leaflet in a patient with anomalous insertion of the papillary muscle.

Supplementary Material 9. A characteristic parasternal long-axis view showing anomalous insertion of the papillary muscle directly into the mitral leaflet without a thin chordae tendineae connection.

Supplementary Material 10. Transesophageal echocardiographic images of a patient with an elongated papillary muscle that inserts into the mitral leaflet via a thin chordae tendineae connection. Flow acceleration due to a thick papillary muscle oriented anteriorly suggests significant left ventricular outflow tract obstruction. Systolic anterior motion of the mitral leaflet is absent.

Supplementary Material 11. Transesophageal echocardiographic images of a patient with an elongated papillary muscle that inserts into the mitral leaflet via a thin chordae tendineae connection. Flow acceleration due to a thick papillary muscle oriented anteriorly suggests significant left ventricular outflow tract obstruction. Systolic anterior motion of the mitral leaflet is absent.

Supplementary Material 12. A magnified parasternal long axis view shows prominent systolic anterior motion of the mitral valve with septal contact.

Supplementary Material 13. Apical view shows an elongated thick papillary muscle oriented anteriorly and in contact with the septal wall during systole.

Supplementary Material 14. In addition to myectomy, the anteriorly displaced papillary muscle was realigned posteriorly using pledged sutures with the adjacent papillary muscle, which resulted in widening of the left ventricular outflow tract.

Supplementary Material 15. Hypertrophied ventricular septal wall contacts the papillary muscle during systole in a patient with midventricular obstruction. Color Doppler flow study clearly demonstrates midventricular flow acceleration.

Supplementary Material 16. Hypertrophied ventricular septal wall contacts the papillary muscle during systole in a patient with midventricular obstruction. Color Doppler flow study clearly demonstrates midventricular flow acceleration.

Supplementary Material 17. Prominent systolic anterior motion of the mitral valve with septal contact and flow acceleration suggestive of left ventricular outflow tract obstruction before mavacamten treatment.

Supplementary Material 18. Prominent systolic anterior motion of the mitral valve with septal contact and flow acceleration suggestive of left ventricular outflow tract obstruction before mavacamten treatment.

Supplementary Material 19. SAM and LVOT obstruction were markedly decreased with mavacamten treatment.

Supplementary Material 20. A magnified apical view showing LVOT obstruction due to elongated papillary muscle.

Supplementary Material 21. A magnified apical view also shows prominent SAM-septal contact.

Supplementary Material 22. Progressive and marked improvement of both SAM and LVOT obstruction due to elongated papillary muscle.

Supplementary Material 23. Progressive and marked improvement of both SAM and LVOT obstruction due to elongated papillary muscle.

Supplementary Material 24. Apical views showing prominent SAM-septal contact and LVOT obstruction in a patient with sigmoid septum before mavacamten treatment.

Supplementary Material 25. Mavacamten successfully relieved SAM and LVOT obstruction in a patient with sigmoid septum.

Supplementary Material 26. Mavacamten successfully relieved SAM and LVOT obstruction in a patient with sigmoid septum.

Supplementary Material 27. Apical views showing mid-ventricular flow acceleration after successful aortic valve replacement.

Supplementary Material 28. Apical views showing mid-ventricular flow acceleration after successful aortic valve replacement.

Supplementary Material 29. A representative case showing SAM-septal contact and LVOT obstruction in a patient with typical apical ballooning.

Supplementary Material 30. Prominent SAM-septal contact with progression of mitral annular calcification.

Supplementary Material 31. Prominent SAM-septal contact with progression of mitral annular calcification.

## Data Availability

No datasets were generated or analysed during the current study.

## Abbreviations

ASH: Asymmetric septal hypertrophy
CMR: Cardiac magnetic resonance imaging
CT: Computed tomography
HCM: Hypertrophic cardiomyopathy
LV: Left ventricular
LVOT: Left ventricular outflow tract
LVOTO: Left ventricular outflow tract obstruction
MAC: Mitral annular calcification
SAM: Systolic anterior motion of the mitral leaflet
SAVR: Surgical aortic valve replacement
TAVI: Transcatheter aortic valve implantation
TEE: Transesophageal echocardiography
