# Correction: Update on left ventricular outflow tract obstruction

**DOI:** 10.1186/s44348-025-00056-3

**Published:** 2025-07-26

**Authors:** Jae‑Kwan Song, Byung Joo Sun, Dae‑Hee Kim, Sung Ho Jung

**Affiliations:** 1 Department of Cardiology, Asan Medical Center, University of Ulsan College of Medicine, Seoul, Republic of Korea; 2 Department of Cardiothoracic Surgery, Asan Medical Center, University of Ulsan College of Medicine, Seoul, Republic of Korea

**Correction: J Cardiovasc Imaging 33, 6 (2025)**

**https://doi.org/10.1186/s44348-025-00049-2**

Following publication of the original article, a minor error was found in the legend of Fig. 5, the differences have been marked in bold.

The incorrect legend of Fig. 5:

Representative cases of various surgical techniques for elongated mitral leaflets in patients with left ventricular outflow tract obstruction. (A, B, **F, G**) Mitral leaflet plication in addition to myectomy is the most frequently performed technique. (**C, D, H, I)** Edge-to-edge repair or (**E**, J) mitral valve replacement is occasionally conducted depending on patient condition. Top and bottom panels show preoperative and postoperative images, respectively.

The correct legend of Fig. 5:

Representative cases of various surgical techniques for elongated mitral leaflets in patients with left ventricular outflow tract obstruction. (A, B, **C, D**) Mitral leaflet plication in addition to myectomy is the most frequently performed technique. (**E, F, G, H**) Edge-to-edge repair or (**I**, J) mitral valve replacement is occasionally conducted depending on patient condition. Top and bottom panels show preoperative and postoperative images, respectively.

The original paper has been updated.

